# A novel algorithmic multi-attribute decision-making framework for solar panel selection using modified aggregations of cubic intuitionistic fuzzy hypersoft set

**DOI:** 10.1016/j.heliyon.2024.e36508

**Published:** 2024-08-22

**Authors:** Muhammad Sajid, Khuram Ali Khan, Atiqe Ur Rahman, Sanaa A. Bajri, Alhanouf Alburaikan, Hamiden Abd El-Wahed Khalifa

**Affiliations:** a Department of Mathematics, University of Sargodha, Sargodha, 40100, Pakistan; b Department of Mathematics, University of Management and Technology, Lahore, 54000, Pakistan; c Department of Mathematical Sciences, College of Science, Princess Nourah bint Abdulrahman University, P.O. Box 84428, Riyadh, 11671, Saudi Arabia; d Department of Mathematics, College of Science, Qassim University, Buraydah, 51452, Saudi Arabia

**Keywords:** Fuzzy set, Cubic set, Cubic soft set, Solar energy, Optimization, Decision making

## Abstract

To address the shortcomings of the cubic intuitionistic fuzzy sets (CIFSs) for the entitlement of multi-argument approximate function, the cubic intuitionistic fuzzy hypersoft set (Ω-set) is an emerging study area. This type of setting associates the sub-parametric tuples with the collection of CIFSs. Categorizing the evaluation of parameters into their corresponding sub-parametric values based on non-overlapping sets has significance in decision making and optimization related situations. Some operations of Ω-set are proposed in this study, along with certain practical features. We provide the complement, P-order, and R-order subsets, P-union ($\cup_{P}$), R-union ($\cup_{R}$), P-intersection ($\cap_{P}$) and R-intersection ($\cap_{R}$) of Ω-sets. The internal cubic intuitionistic fuzzy hypersoft set ($\Omega_{I}$-set) and the external cubic intuitionistic fuzzy hypersoft set ($\Omega_{E}$-set) are also proposed in this paper, which will aid researchers in applying this new theory to other areas of study. We show a few examples in this context and look into some more aspects of $\cup_{P}$, $\cup_{R}$, $\cap_{P}$ and $\cap_{R}$ of $\Omega_{I}$-sets and $\Omega_{E}$-sets. Arguments for a few significant theorems about $\Omega_{I}$-sets and $\Omega_{E}$-sets are also presented. Lastly, an algorithm is presented that assists decision-makers in evaluating appropriate solar panels to establish solar plants. The proposed algorithm uses the idea of $\cup_{P}$ and $\cup_{R}$ for two Ω-sets constructed based on expert opinions of decision makers.

## 1 Introduction

One of the phenomena that we use the most in our daily lives is decision making (DM). To reach the ultimate conclusion, almost all decisions require several phases, some of which may be unclear. The decision-maker must incorporate the preferences for handling uncertainty in the analysis because if the assessment is carried out without addressing the uncertainties in the data, the related outcomes would be quite ambiguous. To tackle this problem, Zadeh [1] presented the idea of the fuzzy set (FS), which has subsequently gained widespread application. In a variety of real-world domains like social science, environmental science, engineering, and economics. Additional FS extensions have been developed and studied, including the intuitionistic fuzzy set (IFS) [2], the interval-valued intuitionistic fuzzy set (IVIFS) [3], linguistic IVIFS [4], complex IFS [5] and complex IVIFS [6]. A study has been done in FS, where decision-makers assess the object by merely expressing the degrees to which they are partial to it; they are unable to represent the non-preferences. Each alternative or object is represented as a pair of membership and non-membership in IFSs or IVIFSs, with the sum of their degrees always less than or equal to one.

In 1999, Molodtsov [7] presented the idea of soft sets (SS). Maji et al. [8], [9] subsequently presented numerous additional procedures on SSs. The study of hybrid models, which combine SS with other models, has been developed in the meantime. Examples of these include fuzzy SSs (FSSs) [10], [11], [12], [13], interval-valued FSSs (IVFSSs) [14], [15], intuitionistic FSSs (IFSSs) [16], [17], generalized FSSs [18], generalized IFSSs [19], etc.

In 2012, Jun et al. [20] introduced the idea of a cubic set (CS) by merging the theory of FS with the interval-valued fuzzy set (IVFS) theory. They looked at the complement of CSs, $\cup_{P}$, $\cup_{R}$, $\cap_{P}$, $\cap_{R}$, and other relevant properties of CSs. The field of CS theory has experienced a rapid increase in research recently. As an illustration, Muhiuddin and Al-Roqi [21] introduced the cubic soft set (CSS), a combination of CS and SS, and applied it to BCK/BCI algebras. The degree of rejection or non-membership plays an equal part throughout the performance analysis of any DM problem, even though CSs only considered the acceptance region. Therefore, in light of all of this, Jun [22] proposed the concept of cubic intuitionistic fuzzy set (CIFS), a hybrid set developed by fusing the elements of IVIFSs and IFSs and applying it in BCK/BCI algebras. The same author proposed the concept of a cubic interval-valued intuitionistic fuzzy set (CIVIFS) [23] and discussed its important applications in BCK/BCI algebra. As a generalization of the IFSs and IVIFSs, CIFS is a powerful and useful tool for describing imprecise information. Garg and Kaur [24] further define the $\cap_{P}$, $\cap_{R}$, $\cup_{P}$, $\cup_{R}$, and internal (external) CIFSs based on their fundamental property, and they also introduced several aggregation operators (AOs) for CIFSs [25]. The same authors examined many approaches, such as distance measures [26] and the TOPSIS method [27], for solving decision making problems (DMPs) in the CIFS environment. Recently, Faizi et al. [28] offered an application example that solves an MCDM problem to show the viability of the suggested operations on CIFSs. The idea of the SS was expanded to the hypersoft set (HSS) by Smarandache [29], who did this by converting the soft approximate function into a multi-argument approximate function. FSS environments cannot be used to tackle the problem if an attribute is more than one and is further split. Consequently, a novel setting, known as the fuzzy hypersoft set (FHSS) [30], was required to find a new method of solving such issues. They tackled a DM problem successfully implementing the FHSSs with Roy and Maji's technique. The hybrid form of interval and exact values may be a better way for the decision maker to express his preference in the complex DM dilemma. A cubic intuitionistic fuzzy soft set (CIFSS) was, therefore, developed by Saqlain et al. [31], concurrently defining two components: an IFS and an IVIFS. As a result, the CIFSS handles the alternative's truth value and falseness value over their corresponding intervals jointly. Rahman et al. [32] investigated the interval-valued FHSS (IVFHSS), which is an emerging field of study aimed at addressing the limitations of IVFSSs in handling multi-argument approximate functions (maaf). The IVFHSS has been studied by several scholars, but the work of investigators [33], [34], [35], [36] has been reported significantly as they have applied this concept in various DM scenarios.

Since solar energy is dependent on the sun, a plentiful and continuously regenerated resource, it is seen as an environmentally friendly form of energy. Unlike fossil fuels like coal and oil, we do not consume any limited supplies or release any toxic contaminants into the environment when we use solar energy to generate power. Using solar energy instead of fossil fuels can help us cut down on greenhouse gas emissions considerably. When solar power is generated, very little greenhouse gas is released into the atmosphere, mostly during the solar panel production procedure. Solar panels provide electricity without releasing contaminants after they are deployed. By reducing the rate of global warming, a decline in emissions contributes to the mitigation of climate change. In this effort, solar energy is essential since it offers a clean and sustainable substitute for fossil fuels, lowering the carbon footprint linked to the production of power. We can contribute to a more sustainable future for ourselves and future generations by switching to solar power and other alternative power providers.

There are several uncertainties when choosing solar panels, which may affect your choice. First, new panel types and features are always being introduced by market forces and technological breakthroughs, making it difficult to predict which technology will ultimately provide the highest level of value and performance. Furthermore, there are unknowns about how well solar panels will hold up in various circumstances and over time due to supplier differences in effectiveness, robustness, and dependability. The DM process is made more complex by elements including startup expenses maintenance needs, and integration with current infrastructure. To get the best results in terms of energy generation, cost-effectiveness, and long-term viability balancing these uncertainties necessitates thoughtful assessment of both short- and long-term repercussions.

Researchers have put forth significant endeavors to use various computational frameworks to analyze solar panel selection (SPS) to address these kinds of uncertainty. For example, Pythagorean fuzzy-based operators were employed by Rani et al. [37] to evaluate SPS performance. For the evaluation of SPS, Ihsan et al. [38] and Akram et al. [39] used the concepts of FHS and Fermatean FSS combined with multi-decisive settings. In their discussion of SPS, Raja et al. [40] utilized aggregation operators with generalized N-SS. To address the uncertainties associated with SPS, Tysüz and Kahraman [41], Ziemba and Szaja [42], and Arman and Kundakc [43] used various DM techniques. Jafar et al. [44] formulated trigonometric similarity measures for generalized hypersoft set and discussed the evaluation of renewable energy source selection using these formulations. Similarly, Saqlain et al. [45] integrated the similarity and distance measures with TOPSIS of the generalized hypersoft set to the evaluation of sustainable green security systems. Riaz et al. [46] employed new techniques for the evaluation of renewable energy sources based on distance and entropy measures, and Einstein averaging aggregation operators of bipolar cubic fuzzy sets. The development of hybrid structures that combine HSS and CIFS has yet to be previously investigated. By breaking each attribute down into its parts, this approach can lead to a deeper understanding of DM attributes and improved efficiency and effectiveness of DM processes. The cubic intuitionistic fuzzy hypersoft set (Ω-set), a hybrid fuzzy structure, was developed by Saeed et al. [47] in response to the need for a versatile analysis tool that could be fully evaluated at the sub-attribute level. A strong addition to FS theory, the CIFS combines two membership functions, i.e., membership and non-membership, with two fuzzy intervals, that is, membership and non-membership intervals that either contain or do not contain membership and non-membership functions. This makes it easier to represent ambiguity and uncertainty in DM processes. Conversely, the primary focus of HSS theory is a sub-attribute analysis of attribute-based data from SS theory. These two frameworks were combined to develop the Ω-set, which offers an accurate and adaptable method for decision analysis. Thus, it can be concluded that the development of Ω-set is meant to cope with the following challenges:

1. The membership and non-membership grades in IFS and IFSS are typically real-valued, with the requirement that their sums fall between [0,1] to account for uncertainties and vagueness, respectively. This forces the decision-makers to rely on one another, but it is much more practical to give them precise ranges to choose from so they can make wise decisions.
2. For a consistent and dependable DM process, it is crucial to take into account all of the parameters and their sub-parametric values. Just taking into account a small number of parameters and ignoring their respective sub-parametric values can cast doubt on the process.

The versatile approximate function of the suggested Ω-set, with its multi-argument domain (HSS settings) for handling the second problem and its vast range (CIFS) for handling the first, allows it to readily manage these challenges. The salient contributions of the study are outlined as:

1. Combining the concepts of CIFS and HSS, an adaptable theoretical structure called Ω-set is developed. The first one aims to give a more comprehensive understanding of membership and non-membership grades, as well as their corresponding interval-valued ranges. On the other hand, the latter offers an approximate function with a multi-argument domain to handle attribute-valued non-overlapping sets. Therefore, the incorporation of these relevant concepts increases the versatility of Ω-set in decision analysis.
2. The set operations $\cup_{P}$, $\cup_{R}$ and the intersection between the Ω-set family have been introduced. The basic features of the internal (or external) Ω-sets have been shown by several results, which also demonstrate that their union or intersection need not be internal (or external) Ω-sets. Additionally, a few restrictions on the two internal (or external) Ω-sets are provided, according to which an internal or external Ω-set is the $\cup_{P}$ and $\cup_{R}$ or intersection of the two Ω-sets.
3. To solve a multi-attribute decision making (MADM) problem, an effective algorithm is presented using the suggested set operations of Ω-set. An analysis of a prototype case study that helps an agriculturist assess solar panels that are optimized helps to explain this algorithm.
4. The suggested strategy's effectiveness and adaptability are evaluated by employing a thorough comparison with a few pertinent specified approaches.

The following is how the study is set up: A few selected preliminary definitions that are necessary for introducing Ω-set are provided in Section 2. In Section 3, the concepts of Ω-set, $\Omega_{I}$-set, and $\Omega_{E}$-set are presented. Additionally, the $\cup_{P}$ and $\cup_{R}$, $\cap_{P}$ and $\cap_{R}$, and complement of Ω-sets are defined. Several significant characteristics of the proposed sets that describe the intrinsic behavior are also covered. The basic properties of $\Omega_{I}$-sets and $\Omega_{E}$-sets are discussed, along with the proofs necessary to support their use. In Section 4, the application is demonstrated through the development of a MADM algorithm utilizing the $\cup_{P}$ and $\cup_{R}$ of Ω-sets. The conclusion section then summarizes the paper's key findings and upcoming projects.

## 2 Preliminaries

This section consists of some elementary definitions that are necessary for understanding the concept of this paper. Let X be an initial universe set and let **E** be a set of parameters and G⊆E. A FS A on X is characterized by mapping $\mu_{A}:X⟶[0,1]$. For each x∈X, the value $\mu_{A}(x)$ is called the membership grade of *x* to $\mu_{A}$. A function ${\overset{˜}{\mu }}_{A}:X⟶P([0,1])$ is called the IVFS on X, where P([0,1]) is the collection of sub-intervals of [0,1]. For every x∈X, the value ${\overset{˜}{\mu }}_{A}(x)=[{\overset{˜}{\mu }}_{A}^{L}(x),{\overset{˜}{\mu }}_{A}^{U}(x)]$ is the membership grade of *x* to ${\overset{˜}{\mu }}_{A}$, where ${\overset{˜}{\mu }}_{A}^{L}:X⟶[0,1]$ and ${\overset{˜}{\mu }}_{A}^{U}:X⟶[0,1]$ are both FSs known as lower and upper FS on X respectively. Combination of IVFS and FS on X in the form $\left\{\langle x,{\overset{˜}{\mu }}_{A}(x),\mu_{A}(x)\rangle :x\in X\right\}$ known as CS on X. $C^{X}$ represents the collection of all CSs on X.

Definition 2.1 **[2]**

An IFS B on X, is given by

$$B=\{\langle x,\sigma_{B}(x),\varsigma_{B}(x)\rangle :x\in X\}$$

where $\sigma_{B}:X⟶[0,1]$ and $\varsigma_{B}:X⟶[0,1]$ with condition $0\leq \sigma_{B}(x)+\varsigma_{B}(x)\leq 1$. Also, $\sigma_{B}(x)\in [0,1]$ and $\varsigma_{B}(x)\in [0,1]$ represent the degrees of membership and non-membership of x∈X respectively. The value $\pi_{B}(x)=1-\sigma_{B}(x)-\varsigma_{B}(x)$ represents the level of hesitancy for membership of element x∈X. The IFS can simply be written as $\left(\sigma_{B},\varsigma_{B}\right)$. The collection of all IFSs over X will be denoted by F(X).

Definition 2.2 **[2]**

Let $B=(\sigma_{B},\varsigma_{B}),\;\text{and}\;D=(\sigma_{D},\varsigma_{D})$ be two IFSs. The following expressions are thus defined as

1. B⊆D if $\sigma_{B}(x)\leq \sigma_{D}(x)$ and $\varsigma_{B}(x)\geq \varsigma_{D}(x)$ for all x∈X;
2. B=D if and only if B⊆D and D⊆B;
3. $B^{c}=\{\langle x,\varsigma_{B}(x),\sigma_{B}(x)\rangle :x\in X\}$;
4. $B\cup D=(max(\sigma_{B}(x),\sigma_{D}(x)),min(\varsigma_{B}(x),\varsigma_{D}(x)))=B\vee D$;
5. $B\cap D=(min(\sigma_{B}(x),\sigma_{D}(x)),max(\varsigma_{B}(x),\varsigma_{D}(x)))=B\wedge D$.

The membership functions in IFSs are depicted as pointed numbers. But if a decision must be made using interval numbers, Atanassov and Gargov [3] expanded the idea of IFSs to develop an IVIFS, which can be defined as follows:

Definition 2.3 **[3]**

An IVIFS, $\overset{˜}{A}$ on X is given as

$$\overset{˜}{A}=\{\langle x,{\overset{˜}{\sigma }}_{\overset{˜}{A}}(x),{\overset{˜}{\varsigma }}_{\overset{˜}{A}}(x)\rangle :x\in X\}$$

where ${\overset{˜}{\sigma }}_{\overset{˜}{A}}:X⟶P([0,1])$ and ${\overset{˜}{\varsigma }}_{\overset{˜}{A}}:X⟶P([0,1])$ such that ${\overset{˜}{\sigma }}_{\overset{˜}{A}}(x)=[\sigma_{\overset{˜}{A}}^{L}(x),\sigma_{\overset{˜}{A}}^{U}(x)]$ and ${\overset{˜}{\varsigma }}_{\overset{˜}{A}}(x)=[\varsigma_{\overset{˜}{A}}^{L}(x),\varsigma_{\overset{˜}{A}}^{U}(x)]$ represent the membership and non-membership grade of x∈X respectively, with $0\leq \sigma_{\overset{˜}{A}}^{L}(x)\leq \sigma_{\overset{˜}{A}}^{U}(x)\leq 1,0\leq \varsigma_{\overset{˜}{A}}^{L}(x)\leq \varsigma_{\overset{˜}{A}}^{U}(x)\leq 1$ and $\sigma_{\overset{˜}{A}}^{U}(x)+\varsigma_{\overset{˜}{A}}^{U}\leq 1$. The IVIFS can simply be written as $\overset{˜}{A}=({\overset{˜}{\sigma }}_{\overset{˜}{A}},{\overset{˜}{\varsigma }}_{\overset{˜}{A}})$. The collection of all IVIFSs over X will be denoted by $\overset{˜}{F}\left(X\right)$.

We define the join ∨ and meet ∧ operations for two IVIFSs, in X. For $\overset{˜}{A},\overset{˜}{B}\in \overset{˜}{F}\left(X\right)$, we have:

1. $\left(\overset{˜}{A}\vee \overset{˜}{B})=([\text{sup}(\sigma_{\overset{˜}{A}}^{L},\sigma_{\overset{˜}{B}}^{L}),\text{sup}(\sigma_{\overset{˜}{A}}^{U},\sigma_{\overset{˜}{B}}^{U})],[\text{inf}(\varsigma_{\overset{˜}{A}}^{L},\varsigma_{\overset{˜}{B}}^{L}),\text{inf}(\varsigma_{\overset{˜}{A}}^{U},\varsigma_{\overset{˜}{B}}^{U})]\right)$,
2. $\left(\overset{˜}{A}\wedge \overset{˜}{B})=([\text{inf}(\sigma_{\overset{˜}{A}}^{L},\sigma_{\overset{˜}{B}}^{L}),\text{inf}(\sigma_{\overset{˜}{A}}^{U},\sigma_{\overset{˜}{B}}^{U})],[\text{sup}(\varsigma_{\overset{˜}{A}}^{L},\varsigma_{\overset{˜}{B}}^{L}),\text{sup}(\varsigma_{\overset{˜}{A}}^{U},\varsigma_{\overset{˜}{B}}^{U})]\right)$.

Definition 2.4 **[7]**

Let P(X) be the power set of universe set X. A SS $F_{E}$ over X, is defined by a function $f_{E}$ representing a mapping:

$$f_{E}:E⟶P\left(X\right)\;\text{such that}\;f_{E}(x)=\phi \;\text{if}\;x\notin E.$$

In other words, $F_{E}$ is parameterized family of subsets of X. Here, for each x∈E, the set $f_{E}\left(x\right)$ is called the value set of *x* in $F_{E}$. Thus, $F_{E}$ over X can be represented by the set of ordered pairs

$$F_{E}=\{(x,f_{E}(x)):x\in E,f_{E}\left(x\right)\in P\left(X\right)\}.$$

Definition 2.5 **[16]**

An IFSS over X is a pair $\left(\zeta_{G},G\right)$, where $\zeta_{G}$ is an approximate mapping given as

$$\zeta_{G}:E⟶F\left(X\right)\;\text{such that}\;\zeta_{G}(\epsilon )=\phi \;\text{if}\;\epsilon \notin G,$$

G⊆E and F(X) is the collection of all IFSs over X. For each $\epsilon \in E,\zeta_{G}(\epsilon )$ represents the *ϵ*-element of the $\left(\zeta_{G},G\right)$. Where $\zeta_{G}(\epsilon )$ can be written as:

$$\zeta_{G}(\epsilon )=\{\langle x,\mu_{\zeta_{G}(\epsilon )}(x),\nu_{\zeta_{G}(\epsilon )}(x)\rangle :x\in X\}$$

where $\mu_{\zeta_{G}(\epsilon )}(x)$ and $\nu_{\zeta_{G}(\epsilon )}(x)$ are the membership and non-membership degrees of *x* in the F(X), satisfying $0\leq \mu_{\zeta_{G}(\epsilon )}(x)+\nu_{\zeta_{G}(\epsilon )}(x)\leq 1$ for all x∈X. Thus

$$(\zeta_{G},G)=\{(\epsilon ,\zeta_{G}(\epsilon )):\epsilon \in E\}.$$

Definition 2.6 **[20]**

A CS, U on X≠ϕ is a structure U={〈x,A(x),λ(x)〉:x∈X} in which A is an interval-valued fuzzy set (IVF) on X and *λ* is a fuzzy set on X. It is denoted by U=〈A,λ〉. The collection of all CSs is denoted by $C^{X}$.

Definition 2.7 **[22]**

A CIFS, $\overset{˜}{U}$ define over X≠ϕ has a structure given as

$$\overset{˜}{U}=\{\langle x,\overset{˜}{A}(x),\overset{˜}{\lambda }(x)\rangle :x\in X,\overset{˜}{A}(x)\in \overset{˜}{F}\left(X\right),\overset{˜}{\lambda }(x)\in F\left(X\right)\}$$

we denote CIFS by pairs as $\overset{˜}{U}=(\overset{˜}{A},\overset{˜}{\lambda })$, where $\overset{˜}{A}=({\overset{˜}{\sigma }}_{\overset{˜}{A}}=[\sigma_{\overset{˜}{A}}^{L},\sigma_{\overset{˜}{A}}^{U}],{\overset{˜}{\varsigma }}_{\overset{˜}{A}}=[\varsigma_{\overset{˜}{A}}^{L},\varsigma_{\overset{˜}{A}}^{U}])$ and $\overset{˜}{\lambda }=(\sigma_{\overset{˜}{A}},\varsigma_{\overset{˜}{A}})$ are IVIFS and IFS. The family of all CIFSs over X is denoted by ${\overset{˜}{C}}^{X}$.

Definition 2.8 **[31]**

A CIFSS, $\left(J_{G},G\right)$ has an approximate mapping $J_{G}$ given as $J_{G}:E⟶{\overset{˜}{C}}^{X}\;\text{such that}\;J_{G}(\epsilon )=\phi \;\text{if}\;\epsilon \notin G\subseteq E$ For each $\epsilon \in E,J_{G}(\epsilon )$ denotes the *ϵ*-element of the CIFS and expressed as

$$J_{G}(\epsilon )=\{\langle x,\overset{˜}{G}(x),\overset{˜}{\lambda }(x)\rangle :x\in X,\overset{˜}{G}(x)\in \overset{˜}{F}\left(X\right),\overset{˜}{\lambda }(x)\in F\left(X\right)\}.$$

Thus a CIFSS, $\left(J_{G},G\right)$ over X can be expressed by the set of ordered pairs

$$(J_{G},G)=\{(\epsilon ,J_{G}(\epsilon )):\epsilon \in G\subseteq E\}.$$

Definition 2.9 **[29]**

Let $Z_{1},Z_{2},Z_{3},...,Z_{n}$ be *n* sets of parameter with no common elements having the sub-parametric values $z_{1},z_{2},z_{3},...,z_{n}$ of the parameters respectively. The HSS $\Psi_{A}$ on universe set X can be written in the form of pairs as

$$\Psi_{A}=\{(\vartheta_{j},\Psi (\vartheta_{j})):\vartheta_{j}\in Z=\prod_{i=1}^{n}Z_{i}\},$$

where A⊆ZandΨ:Z⟶P(X) such that $\Psi (\vartheta_{j})=\phi \;\text{if}\;\vartheta_{j}\notin A$.

## 3 The notions of Ω-set and its properties

The definition of Ω-set and basic concepts associated with it are covered in this section. The following defintion of Ω-set is modified version of Ω-set discussed by Saeed et al. [47].

Definition 3.1 If $\overset{˜}{F}\left(X\right)$ and F(X) are the collections of all IVIFSs and IFSs on X respectively. Also $z_{i}$ are sub parametric values contained in the sets of parameter $Z_{i}$ for 1≤i≤n respectively, with $Z_{i}\cap Z_{j}=\phi$ for i≠j,1≤i,j≤n. Now Ω-set, (Γ,A) on the universe set X can be expressed as:

$$\left(\Gamma ,A\right)=\{(\vartheta_{j},\Gamma (\vartheta_{j})):\vartheta_{j}\in Z=\prod_{i=1}^{n}Z_{i}\},$$

where $\Gamma :Z⟶{\overset{˜}{C}}^{X}$ such that $\Gamma (\vartheta_{j})=\phi \;\text{if}\;\vartheta_{j}\notin A\subseteq Z$, and *ϕ* is cubic intuitionistic fuzzy empty set. Γ is called the approximate function of Ω-set, (Γ,A). For each $\vartheta_{j}\in A,\Gamma (\vartheta_{j})$ is the set of $\vartheta_{j}$-approximate element of the CIFS. It can be written as:

$$\Gamma (\vartheta_{j})=\{\langle x,{\overset{˜}{A}}_{\Gamma (\vartheta_{j})}(x),{\overset{˜}{\lambda }}_{\Gamma (\vartheta_{j})}(x)\rangle :x\in X,{\overset{˜}{A}}_{\Gamma (\vartheta_{j})}(x)\in \overset{˜}{F}\left(X\right),{\overset{˜}{\lambda }}_{\Gamma (\vartheta_{j})}(x)\in F\left(X\right)\}\;or\;\Gamma (\vartheta_{j})=\{\langle x,\frac{\left[\sigma_{\Gamma (\vartheta_{j})}^{L}(x),\sigma_{\Gamma (\vartheta_{j})}^{U}(x)\right]}{\sigma_{\Gamma (\vartheta_{j})}(x)},\frac{\left[\varsigma_{\Gamma (\vartheta_{j})}^{L}(x),\varsigma_{\Gamma (\vartheta_{j})}^{U}(x)\right]}{\varsigma_{\Gamma (\vartheta_{j})}(x)}\rangle :x\in X\}.$$

If $\pi_{\Gamma (\vartheta_{j})}(x)=1-\sigma_{\Gamma (\vartheta_{j})}(x)-\varsigma_{\Gamma (\vartheta_{j})}(x)$. Hence, $\pi_{\Gamma (\vartheta_{j})}(x)$ represents the level of hesitancy for membership of element x∈X. The collection of all Ω-sets over X is denoted by $\Omega_{\left(\Gamma ,A\right)}$.

Remark 3.2 Some special cases of Ω-set for zero level of hesitancy are summarized as follows:

1. A Ω-set, $\left(\Gamma ,A\right)=\{(\vartheta_{j},\{x,\langle \frac{\left[0,0\right]}{1},\frac{\left[1,1\right]}{0}\rangle \}):\forall \;\vartheta_{j}\in Z\;,x\in X\}$ is denoted by $\overset{¨}{0}$.
2. A Ω-set, $\left(\Gamma ,A\right)=\{(\vartheta_{j},\{x,\langle \frac{\left[1,1\right]}{0},\frac{\left[0,0\right]}{1}\rangle \}):\forall \;\vartheta_{j}\in Z\;,x\in X\}$ is denoted by $\overset{¨}{1}$.
3. A Ω-set, $\left(\Gamma ,A\right)=\{(\vartheta_{j},\{x,\langle \frac{\left[0,0\right]}{0},\frac{\left[1,1\right]}{1}\rangle \}):\forall \;\vartheta_{j}\in Z\;,x\in X\}$ is denoted by $\overset{ˆ}{0}$.
4. A Ω-set, $\left(\Gamma ,A\right)=\{(\vartheta_{j},\{x,\langle \frac{\left[1,1\right]}{1},\frac{\left[0,0\right]}{0}\rangle \}):\forall \;\vartheta_{j}\in Z\;,x\in X\}$ is denoted by $\overset{ˆ}{1}$.

Remark 3.3 For any X≠ϕ, let 1(x)=1,0(x)=0. Then,

$$\left(\Gamma ,A\right)=\{(\vartheta_{j},\{x,\langle \frac{{\overset{˜}{\sigma }}_{\Gamma (\vartheta_{j})}(x)}{1},\frac{{\overset{˜}{\varsigma }}_{\Gamma (\vartheta_{j})}(x)}{0}\rangle \}):\forall \;\vartheta_{j}\in A\subseteq Z\;,x\in X\},\left(\Gamma ,B\right)=\{(\vartheta_{j},\{x,\langle \frac{{\overset{˜}{\sigma }}_{\Gamma (\vartheta_{j})}(x)}{0},\frac{{\overset{˜}{\varsigma }}_{\Gamma (\vartheta_{j})}(x)}{1}\rangle \}):\forall \;\vartheta_{j}\in B\subseteq Z\;,x\in X\},\left(\Gamma ,C\right)=\{(\vartheta_{j},\{x,\langle \frac{{\overset{˜}{\sigma }}_{\Gamma (\vartheta_{j})}(x)}{\frac{1}{2}\left(\sigma_{\Gamma (\vartheta_{j})}^{L}+\sigma_{\Gamma (\vartheta_{j})}^{U}\right)},\frac{{\overset{˜}{\varsigma }}_{\Gamma (\vartheta_{j})}(x)}{\frac{1}{2}\left(\varsigma_{\Gamma (\vartheta_{j})}^{L}+\varsigma_{\Gamma (\vartheta_{j})}^{U}\right)}\rangle \}):\forall \;\vartheta_{j}\in C\subseteq Z\;,x\in X\},$$

are all Ω-sets on X.

Definition 3.4 For any Ω-set, $\left(\Gamma ,A\right)\in \Omega_{\left(\Gamma ,A\right)}$, the score value of (Γ,A) is defined as

$$\text{Sc}[\left(\Gamma ,A\right)]=\frac{1}{3}[\left(\sigma_{\Gamma (\vartheta_{j})}^{L}+\sigma_{\Gamma (\vartheta_{j})}^{U}+\sigma_{\Gamma (\vartheta_{j})}\right)-\left(\varsigma_{\Gamma (\vartheta_{j})}^{L}+\varsigma_{\Gamma (\vartheta_{j})}^{U}+\varsigma_{\Gamma (\vartheta_{j})}\right)],$$

where Sc[(Γ,A)]∈[−1,1]. Score values of Ω-sets, $\overset{¨}{0},\overset{ˆ}{0},\overset{¨}{1},\text{ and }\overset{ˆ}{1}$ given in Remark 3.2 are $\text{Sc}\left(\overset{¨}{0}\right)=-0.333$, $\text{Sc}\left(\overset{ˆ}{0}\right)=-1$, $\text{Sc}\left(\overset{¨}{1}\right)=0.333$, and $\text{Sc}\left(\overset{ˆ}{1}\right)=1$ respectively.

Definition 3.5 For $\left(\Gamma ,A\right)\;\text{and}\;\left(\Theta ,B\right)\in \Omega_{\left(\Gamma ,A\right)}$, where A and B are any two subsets of Z. We define the following as:

1. Equality: (Γ,A)=(Θ,B)⇔A=B and ${\overset{˜}{\sigma }}_{\Gamma \left(\vartheta_{j}\right)}(x)={\overset{˜}{\sigma }}_{\Theta \left(\vartheta_{j}\right)}(x);{\overset{˜}{\varsigma }}_{\Gamma \left(\vartheta_{j}\right)}(x)={\overset{˜}{\varsigma }}_{\Theta \left(\vartheta_{j}\right)}(x)\Leftrightarrow \sigma_{\Gamma \left(\vartheta_{j}\right)}^{L}(x)=\sigma_{\Theta \left(\vartheta_{j}\right)}^{L}(x)$ and $\sigma_{\Gamma \left(\vartheta_{j}\right)}^{U}(x)=\sigma_{\Theta \left(\vartheta_{j}\right)}^{U}(x);\varsigma_{\Gamma \left(\vartheta_{j}\right)}^{L}(x)=\varsigma_{\Theta \left(\vartheta_{j}\right)}^{L}(x)$ and $\varsigma_{\Gamma \left(\vartheta_{j}\right)}^{U}(x)=\varsigma_{\Theta \left(\vartheta_{j}\right)}^{U}(x)\;\forall \;\vartheta_{j}\in Z,x\in X$.
2. *P*-order: $\left(\Gamma ,A\right)\subseteq_{P}\left(\Theta ,B\right)\Leftrightarrow A\subseteq B$ and ${\overset{˜}{\sigma }}_{\Gamma \left(\vartheta_{j}\right)}(x)\subseteq {\overset{˜}{\sigma }}_{\Theta \left(\vartheta_{j}\right)}(x),\sigma_{\Gamma \left(\vartheta_{j}\right)}(x)\leq \sigma_{\Theta \left(\vartheta_{j}\right)}(x);{\overset{˜}{\varsigma }}_{\Gamma \left(\vartheta_{j}\right)}(x)⊇{\overset{˜}{\varsigma }}_{\Theta \left(\vartheta_{j}\right)}(x),\varsigma_{\Gamma \left(\vartheta_{j}\right)}(x)\geq \varsigma_{\Theta \left(\vartheta_{j}\right)}(x)\;\forall \;\vartheta_{j}\in Z,x\in X$.
3. *R*-order: $\left(\Gamma ,A\right)\subseteq_{R}\left(\Theta ,B\right)\Leftrightarrow A\subseteq B$ and ${\overset{˜}{\sigma }}_{\Gamma \left(\vartheta_{j}\right)}(x)\subseteq {\overset{˜}{\sigma }}_{\Theta \left(\vartheta_{j}\right)}(x),\sigma_{\Gamma \left(\vartheta_{j}\right)}(x)\geq \sigma_{\Theta \left(\vartheta_{j}\right)}(x);{\overset{˜}{\varsigma }}_{\Gamma \left(\vartheta_{j}\right)}(x)⊇{\overset{˜}{\varsigma }}_{\Theta \left(\vartheta_{j}\right)}(x),\varsigma_{\Gamma \left(\vartheta_{j}\right)}(x)\leq \varsigma_{\Theta \left(\vartheta_{j}\right)}(x)\;\forall \;\vartheta_{j}\in Z,x\in X$.

Definition 3.6 A Ω-set, (Γ,A) is said to be an internal cubic intuitionistic fuzzy hypersoft set ($\Omega_{I}$-set) or external cubic intuitionistic fuzzy hypersoft set ($\Omega_{E}$-set), accordingly

$$\sigma_{\Gamma \left(\vartheta_{j}\right)}^{L}(x)\leq \sigma_{\Gamma \left(\vartheta_{j}\right)}(x)\leq \sigma_{\Gamma \left(\vartheta_{j}\right)}^{U}(x)\;\;\text{and }\;\;\varsigma_{\Gamma \left(\vartheta_{j}\right)}^{L}(x)\leq \varsigma_{\Gamma \left(\vartheta_{j}\right)}(x)\leq \varsigma_{\Gamma \left(\vartheta_{j}\right)}^{U}(x)\sigma_{\Gamma \left(\vartheta_{j}\right)}(x)\notin (\sigma_{\Gamma \left(\vartheta_{j}\right)}^{L}(x),\sigma_{\Gamma \left(\vartheta_{j}\right)}^{U}(x))\;\;\text{and }\;\;\varsigma_{\Gamma \left(\vartheta_{j}\right)}(x)\notin (\varsigma_{\Gamma \left(\vartheta_{j}\right)}^{L}(x),\varsigma_{\Gamma \left(\vartheta_{j}\right)}^{U}(x)).$$

$\forall \;\vartheta_{j}\in A\subseteq Z,\;x\in X$.

Example 3.7 Suppose the Example 4.2, if $\left(\Gamma ,A\right)=\{(\vartheta_{j},\{x,\langle \frac{{\overset{˜}{\sigma }}_{\Gamma (\vartheta_{j})}(x)}{\sigma_{\Gamma (\vartheta_{j})}(x)},\frac{{\overset{˜}{\varsigma }}_{\Gamma (\vartheta_{j})}(x)}{\varsigma_{\Gamma (\vartheta_{j})}(x)}\rangle \})\}$ and $\left(\Theta ,B\right)=\{(\vartheta_{j},\{x,\langle \frac{{\overset{˜}{\sigma }}_{\Theta (\vartheta_{j})}(x)}{\sigma_{\Theta (\vartheta_{j})}(x)},\frac{{\overset{˜}{\varsigma }}_{\Theta (\vartheta_{j})}(x)}{\varsigma_{\Theta (\vartheta_{j})}(x)}\rangle \})\}\in \Omega_{\left(\Gamma ,A\right)}$ are two Ω-sets on the universal set X.

1. Let ${\overset{˜}{\sigma }}_{\Gamma (\vartheta_{j})}(x)=[0.2,0.6]$, $\sigma_{\Gamma (\vartheta_{j})}(x)=0.3$ and ${\overset{˜}{\varsigma }}_{\Gamma (\vartheta_{j})}(x)=[0.1,0.3]$, $\varsigma_{\Gamma (\vartheta_{j})}(x)=0.15$, $\forall \;\vartheta_{j}\in A\subseteq Z,\;x\in X$. Then (Γ,A) is $\Omega_{I}$-set on X.
2. Let ${\overset{˜}{\sigma }}_{\Theta (\vartheta_{j})}(x)=[0.3,0.5]$, $\sigma_{\Theta (\vartheta_{j})}(x)=0.2$ and ${\overset{˜}{\varsigma }}_{\Theta (\vartheta_{j})}(x)=[0.2,0.4]$, $\varsigma_{\Theta (\vartheta_{j})}(x)=0.45$, $\forall \;\vartheta_{j}\in B\subseteq Z,\;x\in X$. Then (Θ,B) is $\Omega_{E}$-set on X.

### 3.1 Set theoretic operations

In this section, we define the basic set theoretic operations, namely the complement, P(R)-union, and P(R)-intersection of Ω-sets.

Definition 3.8 The complement of a Ω-set,

$$\left(\Gamma ,A\right)=\{(\vartheta_{j},\{x,\langle \frac{{\overset{˜}{\sigma }}_{\Gamma (\vartheta_{j})}(x)}{\sigma_{\Gamma (\vartheta_{j})}(x)},\frac{{\overset{˜}{\varsigma }}_{\Gamma (\vartheta_{j})}(x)}{\varsigma_{\Gamma (\vartheta_{j})}(x)}\rangle \}):\vartheta_{j}\in A\subseteq Z,x\in X\}$$

is denoted by ${\left(\Gamma ,A\right)}^{c}$ and is defined as

$${\left(\Gamma ,A\right)}^{c}=\{(\vartheta_{j},\{x,\langle \frac{{\overset{˜}{\varsigma }}_{\Gamma (\vartheta_{j})}(x)}{\varsigma_{\Gamma (\vartheta_{j})}(x)},\frac{{\overset{˜}{\sigma }}_{\Gamma (\vartheta_{j})}(x)}{\sigma_{\Gamma (\vartheta_{j})}(x)}\rangle \}):\vartheta_{j}\in A\subseteq Z,x\in X\}.$$

Obviously ${\left({\left(\Gamma ,A\right)}^{c}\right)}^{c}=\left(\Gamma ,A\right)$.

Example 3.9 The complement ${\left(\Gamma ,A\right)}^{c}$ of the Ω-set (Γ,A) defined in Table 1 is given in Table 2.

**Table 1:** Tabular form of Cubic Intuitionistic Fuzzy Hypersoft set ${\left(\Gamma ,A\right)}^{c}$.

${\left(\Gamma ,A\right)}^{c}$	*ϑ*_1_	*ϑ*_2_	*ϑ*_3_	*ϑ*_4_
$C_{1}$	$\langle \frac{\left[0.4,0.5\right]}{0.65},\frac{\left[0.3,0.45\right]}{0.3}\rangle$	$\langle \frac{\left[0.2,0.4\right]}{0.55},\frac{\left[0.3,0.55\right]}{0.2}\rangle$	$\langle \frac{\left[0.1,0.3\right]}{0.35},\frac{\left[0.2,0.55\right]}{0.4}\rangle$	$\langle \frac{\left[0.3,0.4\right]}{0.35},\frac{\left[0.25,0.4\right]}{0.6}\rangle$
$C_{2}$	$\langle \frac{\left[0.25,0.4\right]}{0.3},\frac{\left[0.1,0.5\right]}{0.2}\rangle$	$\langle \frac{\left[0.3,0.5\right]}{0.45},\frac{\left[0.2,0.45\right]}{0.35}\rangle$	$\langle \frac{\left[0.2,0.3\right]}{0.1},\frac{\left[0.35,0.6\right]}{0.5}\rangle$	$\langle \frac{\left[0.2,0.35\right]}{0.5},\frac{\left[0.2,0.6\right]}{0.3}\rangle$
$C_{3}$	$\langle \frac{\left[0.1,0.3\right]}{0.25},\frac{\left[0.25,0.5\right]}{0.6}\rangle$	$\langle \frac{\left[0.2,0.4\right]}{0.3},\frac{\left[0.15,0.5\right]}{0.4}\rangle$	$\langle \frac{\left[0.3,0.55\right]}{0.5},\frac{\left[0.2,0.4\right]}{0.25}\rangle$	$\langle \frac{\left[0.2,0.3\right]}{0.25},\frac{\left[0.3,0.65\right]}{0.6}\rangle$
$C_{4}$	$\langle \frac{\left[0.2,0.4\right]}{0.1},\frac{\left[0.3,0.55\right]}{0.7}\rangle$	$\langle \frac{\left[0.3,0.5\right]}{0.25},\frac{\left[0.25,0.4\right]}{0.2}\rangle$	$\langle \frac{\left[0.35,0.4\right]}{0.3},\frac{\left[0.3,0.5\right]}{0.45}\rangle$	$\langle \frac{\left[0.1,0.4\right]}{0.45},\frac{\left[0.4,0.55\right]}{0.5}\rangle$ .

**Table 2:** Tabular form of Ω-sets $\left(\Gamma_{1},A\right),\left(\Gamma_{2},B\right),{\left(\Gamma_{1},A\right)}^{⁎}$ and ${\left(\Gamma_{2},B\right)}^{⁎}$.

X	$\left(\Gamma_{1},A\right)$	$\left(\Gamma_{2},B\right)$	${\left(\Gamma_{1},A\right)}^{⁎}$	${\left(\Gamma_{2},B\right)}^{⁎}$
$C_{1}$	$\langle \frac{\left[0.45,0.6\right]}{0.65},\frac{\left[0.1,0.25\right]}{0.3}\rangle$	$\langle \frac{\left[0.1,0.35\right]}{0.4},\frac{\left[0.4,0.55\right]}{0.35}\rangle$	$\langle \frac{\left[0.45,0.6\right]}{0.4},\frac{\left[0.1,0.25\right]}{0.35}\rangle$	$\langle \frac{\left[0.1,0.35\right]}{0.65},\frac{\left[0.4,0.55\right]}{0.3}\rangle$
$C_{2}$	$\langle \frac{\left[0.2,0.35\right]}{0.15},\frac{\left[0.4,0.65\right]}{0.7}\rangle$	$\langle \frac{\left[0.3,0.45\right]}{0.5},\frac{\left[0.15,0.3\right]}{0.1}\rangle$	$\langle \frac{\left[0.2,0.35\right]}{0.5},\frac{\left[0.4,0.65\right]}{0.1}\rangle$	$\langle \frac{\left[0.3,0.45\right]}{0.15},\frac{\left[0.15,0.3\right]}{0.7}\rangle$

Definition 3.10 If $\left(\Gamma_{1},A\right)\;\text{and}\;\left(\Gamma_{2},B\right)\in \Omega_{\left(\Gamma ,Z\right)}$, are any two Ω-sets on X. We define $\cup_{P}$ $\left(\Gamma_{1},A\right)\cup_{P}\left(\Gamma_{2},B\right)=\left(ϒ,C\right)$, where A and B are any two subsets of Z and C=A∪B⊆Z and

$$ϒ(\vartheta_{j})=\left\{ \begin{array}{cc} \begin{array}{c} \Gamma_{1}(\vartheta_{j})\\ \Gamma_{2}(\vartheta_{j})\\ \Gamma_{1}(\vartheta_{j})\vee_{P}\Gamma_{2}(\vartheta_{j}) \end{array}\; & \begin{array}{c} \text{if}\;\;\vartheta_{j}\in A-B\subseteq Z\\ \text{if}\;\;\vartheta_{j}\in B-A\subseteq Z\\ \text{if}\;\;\vartheta_{j}\in A\cap B\subseteq Z, \end{array} \end{array}\right.$$

whereas $\Gamma_{1}(\vartheta_{j})\vee_{P}\Gamma_{2}(\vartheta_{j})$ is defined as

$$\Gamma_{1}(\vartheta_{j})\vee_{P}\Gamma_{2}(\vartheta_{j})=\{\langle x,\frac{\text{rmax}\left\{{\overset{˜}{\sigma }}_{\Gamma_{1}(\vartheta_{j})}(x),{\overset{˜}{\sigma }}_{\Gamma_{2}(\vartheta_{j})}(x)\right\}}{\text{max}\left\{\sigma_{\Gamma_{1}(\vartheta_{j})}(x),\sigma_{\Gamma_{2}(\vartheta_{j})}(x)\right\}},\frac{\text{rmin}\left\{{\overset{˜}{\varsigma }}_{\Gamma_{1}(\vartheta_{j})}(x),{\overset{˜}{\varsigma }}_{\Gamma_{2}(\vartheta_{j})}(x)\right\}}{\text{min}\left\{\varsigma_{\Gamma_{1}(\vartheta_{j})}(x),\varsigma_{\Gamma_{2}(\vartheta_{j})}(x)\right\}}\rangle :x\in X\}.$$

Definition 3.11 If $\left(\Gamma_{1},A\right)\;\text{and}\;\left(\Gamma_{2},B\right)\in \Omega_{\left(\Gamma ,Z\right)}$, are any two Ω-sets on X. We define $\cap_{P}$ $\left(\Gamma_{1},A\right)\cap_{P}\left(\Gamma_{2},B\right)=\left(\Theta ,C\right)$, where A and B are any two subsets of Z and C=A∪B⊆Z and

$$\Theta (\vartheta_{j})=\left\{ \begin{array}{cc} \begin{array}{c} \Gamma_{1}(\vartheta_{j})\\ \Gamma_{2}(\vartheta_{j})\\ \Gamma_{1}(\vartheta_{j})\wedge_{P}\Gamma_{2}(\vartheta_{j}) \end{array}\; & \begin{array}{c} \text{if}\;\;\vartheta_{j}\in A-B\subseteq Z\\ \text{if}\;\;\vartheta_{j}\in B-A\subseteq Z\\ \text{if}\;\;\vartheta_{j}\in A\cap B\subseteq Z, \end{array} \end{array}\right.$$

whereas $\Gamma_{1}(\vartheta_{j})\wedge_{P}\Gamma_{2}(\vartheta_{j})$ is defined as

$$\Gamma_{1}(\vartheta_{j})\wedge_{P}\Gamma_{2}(\vartheta_{j})=\{\langle x,\frac{\text{rmin}\left\{{\overset{˜}{\sigma }}_{\Gamma_{1}(\vartheta_{j})}(x),{\overset{˜}{\sigma }}_{\Gamma_{2}(\vartheta_{j})}(x)\right\}}{\text{min}\left\{\sigma_{\Gamma_{1}(\vartheta_{j})}(x),\sigma_{\Gamma_{2}(\vartheta_{j})}(x)\right\}},\frac{\text{rmax}\left\{{\overset{˜}{\varsigma }}_{\Gamma_{1}(\vartheta_{j})}(x),{\overset{˜}{\varsigma }}_{\Gamma_{2}(\vartheta_{j})}(x)\right\}}{\text{max}\left\{\varsigma_{\Gamma_{1}(\vartheta_{j})}(x),\varsigma_{\Gamma_{2}(\vartheta_{j})}(x)\right\}}\rangle :x\in X\}.$$

Definition 3.12 If $\left(\Gamma_{1},A\right)\;\text{and}\;\left(\Gamma_{2},B\right)\in \Omega_{\left(\Gamma ,Z\right)}$, are any two Ω-sets on X. We define $\cup_{R}$ $\left(\Gamma_{1},A\right)\cup_{R}\left(\Gamma_{2},B\right)=\left(\overset{˜}{ϒ},C\right)$, where A and B are any two subsets of Z and C=A∪B⊆Z and

$$\overset{˜}{ϒ}(\vartheta_{j})=\left\{ \begin{array}{cc} \begin{array}{c} \Gamma_{1}(\vartheta_{j})\\ \Gamma_{2}(\vartheta_{j})\\ \Gamma_{1}(\vartheta_{j})\vee_{R}\Gamma_{2}(\vartheta_{j}) \end{array}\; & \begin{array}{c} \text{if}\;\;\vartheta_{j}\in A-B\subseteq Z\\ \text{if}\;\;\vartheta_{j}\in B-A\subseteq Z\\ \text{if}\;\;\vartheta_{j}\in A\cap B\subseteq Z, \end{array} \end{array}\right.$$

whereas $\Gamma_{1}(\vartheta_{j})\vee_{R}\Gamma_{2}(\vartheta_{j})$ is defined as

$$\Gamma_{1}(\vartheta_{j})\vee_{R}\Gamma_{2}(\vartheta_{j})=\{\langle x,\frac{\text{rmax}\left\{{\overset{˜}{\sigma }}_{\Gamma_{1}(\vartheta_{j})}(x),{\overset{˜}{\sigma }}_{\Gamma_{2}(\vartheta_{j})}(x)\right\}}{\text{min}\left\{\sigma_{\Gamma_{1}(\vartheta_{j})}(x),\sigma_{\Gamma_{2}(\vartheta_{j})}(x)\right\}},\frac{\text{rmin}\left\{{\overset{˜}{\varsigma }}_{\Gamma_{1}(\vartheta_{j})}(x),{\overset{˜}{\varsigma }}_{\Gamma_{2}(\vartheta_{j})}(x)\right\}}{\text{max}\left\{\varsigma_{\Gamma_{1}(\vartheta_{j})}(x),\varsigma_{\Gamma_{2}(\vartheta_{j})}(x)\right\}}\rangle :x\in X\}.$$

Definition 3.13 If $\left(\Gamma_{1},A\right)\;\text{and}\;\left(\Gamma_{2},B\right)\in \Omega_{\left(\Gamma ,Z\right)}$, are any two Ω-sets on X. We define $\cap_{R}$ $\left(\Gamma_{1},A\right)\cap_{R}\left(\Gamma_{2},B\right)=\left(\overset{˜}{\Theta },C\right)$, where A and B are any two subsets of Z and C=A∪B⊆Z and

$$\overset{˜}{\Theta }(\vartheta_{j})=\left\{ \begin{array}{cc} \begin{array}{c} \Gamma_{1}(\vartheta_{j})\\ \Gamma_{2}(\vartheta_{j})\\ \Gamma_{1}(\vartheta_{j})\wedge_{R}\Gamma_{2}(\vartheta_{j}) \end{array}\; & \begin{array}{c} \text{if}\;\;\vartheta_{j}\in A-B\subseteq Z\\ \text{if}\;\;\vartheta_{j}\in B-A\subseteq Z\\ \text{if}\;\;\vartheta_{j}\in A\cap B\subseteq Z, \end{array} \end{array}\right.$$

whereas $\Gamma_{1}(\vartheta_{j})\wedge_{R}\Gamma_{2}(\vartheta_{j})$ is defined as

$$\Gamma_{1}(\vartheta_{j})\wedge_{R}\Gamma_{2}(\vartheta_{j})=\{\langle x,\frac{\text{rmin}\left\{{\overset{˜}{\sigma }}_{\Gamma_{1}(\vartheta_{j})}(x),{\overset{˜}{\sigma }}_{\Gamma_{2}(\vartheta_{j})}(x)\right\}}{\text{max}\left\{\sigma_{\Gamma_{1}(\vartheta_{j})}(x),\sigma_{\Gamma_{2}(\vartheta_{j})}(x)\right\}},\frac{\text{rmax}\left\{{\overset{˜}{\varsigma }}_{\Gamma_{1}(\vartheta_{j})}(x),{\overset{˜}{\varsigma }}_{\Gamma_{2}(\vartheta_{j})}(x)\right\}}{\text{min}\left\{\varsigma_{\Gamma_{1}(\vartheta_{j})}(x),\varsigma_{\Gamma_{2}(\vartheta_{j})}(x)\right\}}\rangle :x\in X\}.$$

Theorem 3.14 *Let*$\left(\Gamma ,A\right)=\langle \frac{{\overset{˜}{\sigma }}_{\Gamma }}{\sigma_{\Gamma }},\frac{{\overset{˜}{\varsigma }}_{\Gamma }}{\varsigma_{\Gamma }}\rangle$*be* Ω*-set on* X *which is not an* $\Omega_{I}$*-set. Then there exist an* x∈X *such that* $\sigma_{\Gamma }(x)\notin (\sigma_{\Gamma \left(\vartheta_{j}\right)}^{L}(x),\sigma_{\Gamma \left(\vartheta_{j}\right)}^{U}(x))$ *and* $\varsigma_{\Gamma }(x)\notin (\varsigma_{\Gamma \left(\vartheta_{j}\right)}^{L}(x),\varsigma_{\Gamma \left(\vartheta_{j}\right)}^{U}(x))$*.*

Proof Straightforward. □

This theorem tells us that if (Γ,A) is a Ω-set which is not $\Omega_{I}$-set then some x∈X must exist that satisfy the condition of $\Omega_{E}$-set. See definitions of $\Omega_{I}$-set and $\Omega_{E}$-set in Definition 3.6.

Theorem 3.15 *Let*$\left(\Gamma ,A\right)=\langle \frac{{\overset{˜}{\sigma }}_{\Gamma }}{\sigma_{\Gamma }},\frac{{\overset{˜}{\varsigma }}_{\Gamma }}{\varsigma_{\Gamma }}\rangle$*be both an*$\Omega_{I}$*-set and*$\Omega_{E}$*-set*$\forall \;\vartheta_{j}\in A\subseteq Z\;\text{and}\;x\in X$*, then*$\sigma_{\Gamma (\vartheta_{j})}(x)\in U_{\sigma }\cup L_{\sigma }$*and*$\varsigma_{\Gamma (\vartheta_{j})}(x)\in U_{\varsigma }\cup L_{\varsigma }$*, where*$U_{\sigma }(x)=\{\sigma_{\Gamma \left(\vartheta_{j}\right)}^{U}(x):x\in X\}$*,*$L_{\sigma }(x)=\{\sigma_{\Gamma \left(\vartheta_{j}\right)}^{L}(x):x\in X\}$*and*$U_{\varsigma }(x)=\{\varsigma_{\Gamma \left(\vartheta_{j}\right)}^{U}(x):x\in X\}$*,*$L_{\varsigma }(x)=\{\varsigma_{\Gamma \left(\vartheta_{j}\right)}^{L}(x):x\in X\}$*.*

Proof Since (Γ,A) is both $\Omega_{I}$-set and $\Omega_{E}$-set. So, we have

$$\sigma_{\Gamma \left(\vartheta_{j}\right)}^{L}(x)\leq \sigma_{\Gamma \left(\vartheta_{j}\right)}(x)\leq \sigma_{\Gamma \left(\vartheta_{j}\right)}^{U}(x)\;\;\text{and }\;\;\sigma_{\Gamma \left(\vartheta_{j}\right)}(x)\notin (\sigma_{\Gamma \left(\vartheta_{j}\right)}^{L}(x),\sigma_{\Gamma \left(\vartheta_{j}\right)}^{U}(x))\varsigma_{\Gamma \left(\vartheta_{j}\right)}^{L}(x)\leq \varsigma_{\Gamma \left(\vartheta_{j}\right)}(x)\leq \varsigma_{\Gamma \left(\vartheta_{j}\right)}^{U}(x)\;\;\text{and }\;\;\varsigma_{\Gamma \left(\vartheta_{j}\right)}(x)\notin (\varsigma_{\Gamma \left(\vartheta_{j}\right)}^{L}(x),\varsigma_{\Gamma \left(\vartheta_{j}\right)}^{U}(x)).\text{Thus},\;\;\;\;\;\;\;\;\;\;\;\sigma_{\Gamma \left(\vartheta_{j}\right)}(x)=\sigma_{\Gamma \left(\vartheta_{j}\right)}^{L}(x)\;\text{or}\;\;\sigma_{\Gamma \left(\vartheta_{j}\right)}^{U}(x)\;\;\text{and}\;\;\varsigma_{\Gamma \left(\vartheta_{j}\right)}(x)=\varsigma_{\Gamma \left(\vartheta_{j}\right)}^{L}(x)\;\text{or}\;\;\varsigma_{\Gamma \left(\vartheta_{j}\right)}^{U}(x).\text{Hence},\;\;\;\;\;\;\;\;\;\;\;\sigma_{\Gamma \left(\vartheta_{j}\right)}(x)\in U_{\sigma }\cup L_{\sigma }\text{ and }\varsigma_{\Gamma \left(\vartheta_{j}\right)}(x)\in U_{\varsigma }\cup L_{\varsigma }.$$

 □

This theorem depicts that if (Γ,A) is a Ω-set which both $\Omega_{I}$-set and $\Omega_{E}$-set then membership degree $\sigma_{\Gamma (\vartheta )}(x)$ of all elements of the universe set is the union of $U_{\varsigma }$ and $L_{\varsigma }$ (i.e. membership degree of all elements of the universe set either upper limit or lower limit of membership interval.

Theorem 3.16 *Let*(Γ,A)*be a* Ω*-set in* X*. If* (Γ,A) *is* $\Omega_{I}$*-set (respectively,* $\Omega_{E}$*-set), then* ${\left(\Gamma ,A\right)}^{c}$ *is also* $\Omega_{I}$*-set (respectively,* $\Omega_{E}$*-set).*

Proof Since $\left(\Gamma ,A\right)=\langle \frac{{\overset{˜}{\sigma }}_{\Gamma }}{\sigma_{\Gamma }},\frac{{\overset{˜}{\varsigma }}_{\Gamma }}{\varsigma_{\Gamma }}\rangle$ is $\Omega_{I}$-set (respectively, $\Omega_{E}$-set). So $\forall \;\vartheta_{j}\in A\subseteq Z,\;x\in X$, we have:

$$\sigma_{\Gamma \left(\vartheta_{j}\right)}^{L}(x)\leq \sigma_{\Gamma \left(\vartheta_{j}\right)}(x)\leq \sigma_{\Gamma \left(\vartheta_{j}\right)}^{U}(x)\;\;\text{and }\;\;\varsigma_{\Gamma \left(\vartheta_{j}\right)}^{L}(x)\leq \varsigma_{\Gamma \left(\vartheta_{j}\right)}(x)\leq \varsigma_{\Gamma \left(\vartheta_{j}\right)}^{U}(x)(\text{respectively,}\;\;\;\sigma_{\Gamma \left(\vartheta_{j}\right)}(x)\notin (\sigma_{\Gamma \left(\vartheta_{j}\right)}^{L}(x),\sigma_{\Gamma \left(\vartheta_{j}\right)}^{U}(x))\;\;\text{and }\;\;\varsigma_{\Gamma \left(\vartheta_{j}\right)}(x)\notin (\varsigma_{\Gamma \left(\vartheta_{j}\right)}^{L}(x),\varsigma_{\Gamma \left(\vartheta_{j}\right)}^{U}(x))).\text{Hence}\;\;\;\;\;\;\;{\left(\Gamma ,A\right)}^{c}=\langle \frac{{\overset{˜}{\varsigma }}_{\Gamma }}{\varsigma_{\Gamma }},\frac{{\overset{˜}{\sigma }}_{\Gamma }}{\sigma_{\Gamma }}\rangle \;\;\text{is also }\Omega_{I}\text{-set (respectively, }\Omega_{E}\text{-set).}$$

 □

This theorem shows that if (Γ,A) is a Ω-set which satisfies the conditions of $\Omega_{I}$-set (or $\Omega_{E}$-set) Then its complement also satisfies the conditions of $\Omega_{I}$-set ($\Omega_{E}$-set).

### 3.2 Significant results of $\Omega_{I}$-set and $\Omega_{E}$-set

Theorem 3.17 *Since*$\left(\Gamma_{i},A_{i}\right)=\langle \frac{{\overset{˜}{\sigma }}_{\Gamma_{i}}}{\sigma_{\Gamma_{i}}},\frac{{\overset{˜}{\varsigma }}_{\Gamma_{i}}}{\varsigma_{\Gamma_{i}}}\rangle \;i\in \Lambda$*be a family of*$\Omega_{I}$*-set in*X*. Then*$\cup_{P}$*and*$\cap_{P}$*of*$\left(\Gamma_{i},A_{i}\right)$*are also*$\Omega_{I}$*-set in*X*.*

Proof Since $\left(\Gamma_{i},A_{i}\right)=\langle \frac{{\overset{˜}{\sigma }}_{\Gamma_{i}}}{\sigma_{\Gamma_{i}}},\frac{{\overset{˜}{\varsigma }}_{\Gamma_{i}}}{\varsigma_{\Gamma_{i}}}\rangle$ is $\Omega_{I}$-set. So for each i∈Λ we have:

$$\sigma_{\Gamma_{i}}^{L}\leq \sigma_{\Gamma_{i}}\leq \sigma_{\Gamma_{i}}^{U}\;\;\text{and }\;\;\varsigma_{\Gamma_{i}}^{L}\leq \varsigma_{\Gamma_{i}}\leq \varsigma_{\Gamma_{i}}^{U}⟹{\left(\underset{i\in \Lambda }{⋁}\sigma_{\Gamma_{i}}\right)}^{L}\leq (\underset{i\in \Lambda }{⋁}\sigma_{\Gamma_{i}})\leq {\left(\underset{i\in \Lambda }{⋁}\sigma_{\Gamma_{i}}\right)}^{U}\;\;\text{and }\;\;{\left(\underset{i\in \Lambda }{⋁}\varsigma_{\Gamma_{i}}\right)}^{L}\leq (\underset{i\in \Lambda }{⋁}\varsigma_{\Gamma_{i}})\leq {\left(\underset{i\in \Lambda }{⋁}\varsigma_{\Gamma_{i}}\right)}^{U}{\left(\underset{i\in \Lambda }{⋀}\sigma_{\Gamma_{i}}\right)}^{L}\leq (\underset{i\in \Lambda }{⋀}\sigma_{\Gamma_{i}})\leq {\left(\underset{i\in \Lambda }{⋀}\sigma_{\Gamma_{i}}\right)}^{U}\;\;\text{and }\;\;{\left(\underset{i\in \Lambda }{⋀}\varsigma_{\Gamma_{i}}\right)}^{L}\leq (\underset{i\in \Lambda }{⋀}\varsigma_{\Gamma_{i}})\leq {\left(\underset{i\in \Lambda }{⋀}\varsigma_{\Gamma_{i}}\right)}^{U}⟹(\underset{i\in \Lambda }{⋁}\sigma_{\Gamma_{i}})\in (\underset{\begin{array}{c} P\\ i\in \Lambda \end{array}}{⋃}{\overset{˜}{\sigma }}_{\Gamma_{i}})\;\;\;\;\;\;\;\text{and }\;\;(\underset{i\in \Lambda }{⋁}\varsigma_{\Gamma_{i}})\in (\underset{\begin{array}{c} P\\ i\in \Lambda \end{array}}{⋃}{\overset{˜}{\varsigma }}_{\Gamma_{i}})(\underset{i\in \Lambda }{⋀}\sigma_{\Gamma_{i}})\in (\underset{\begin{array}{c} P\\ i\in \Lambda \end{array}}{⋂}{\overset{˜}{\sigma }}_{\Gamma_{i}})\;\;\;\;\;\;\;\text{and }\;\;(\underset{i\in \Lambda }{⋀}\varsigma_{\Gamma_{i}})\in (\underset{\begin{array}{c} P\\ i\in \Lambda \end{array}}{⋂}{\overset{˜}{\varsigma }}_{\Gamma_{i}}).$$

Hence ${⋃}_{P}\left(\Gamma_{i},A_{i}\right)$ and ${⋂}_{P}\left(\Gamma_{i},A_{i}\right)$ is also $\Omega_{I}$-set in X. □

This theorem provides important information about the special type of union and intersection of Ω-sets that are the $\cup_{P}$ and $\cap_{P}$ of the collection of $\Omega_{I}$-sets are also $\Omega_{I}$-sets. The following example will demonstrate that $\cup_{P}$ and $\cap_{P}$ of $\Omega_{E}$-sets are not always $\Omega_{E}$-sets.

Example 3.18 Let $\left(\Gamma_{1},A\right)=\langle \frac{{\overset{˜}{\sigma }}_{\Gamma_{1}}}{\sigma_{\Gamma_{1}}},\frac{{\overset{˜}{\varsigma }}_{\Gamma_{1}}}{\varsigma_{\Gamma_{1}}}\rangle$ and $\left(\Gamma_{2},B\right)=\langle \frac{{\overset{˜}{\sigma }}_{\Gamma_{2}}}{\sigma_{\Gamma_{2}}},\frac{{\overset{˜}{\varsigma }}_{\Gamma_{2}}}{\varsigma_{\Gamma_{2}}}\rangle \in \Omega_{\left(\Gamma ,Z\right)}$ be two $\Omega_{E}$-sets on the universal set X with ${\overset{˜}{\sigma }}_{\Gamma_{1}}=[0.1,0.4],\sigma_{\Gamma_{1}}=0.45;{\overset{˜}{\varsigma }}_{\Gamma_{1}}=[0.15,0.3],\varsigma_{\Gamma_{1}}=0.4$ and ${\overset{˜}{\sigma }}_{\Gamma_{2}}=[0.2,0.5],\sigma_{\Gamma_{2}}=0.15;{\overset{˜}{\varsigma }}_{\Gamma_{2}}=[0.3,0.45],\varsigma_{\Gamma_{2}}=0.2$. Now

1. Let $\left(\Gamma_{1},A\right)\cup_{P}\left(\Gamma_{2},B\right)=\left(\Gamma_{3},C\right)$ with ${\overset{˜}{\sigma }}_{\Gamma_{3}}=[0.2,0.5],\sigma_{\Gamma_{3}}=0.45;{\overset{˜}{\varsigma }}_{\Gamma_{3}}=[0.3,0.45],\varsigma_{\Gamma_{3}}=0.4$ which is not $\Omega_{E}$-set on X.
2. Let $\left(\Gamma_{1},A\right)\cap_{P}\left(\Gamma_{2},B\right)=\left(\Gamma_{4},C\right)$ with ${\overset{˜}{\sigma }}_{\Gamma_{4}}=[0.1,0.4],\sigma_{\Gamma_{4}}=0.15;{\overset{˜}{\varsigma }}_{\Gamma_{4}}=[0.15,0.3],\varsigma_{\Gamma_{4}}=0.2$ which is not $\Omega_{E}$-set on X.

In the example below, we will demonstrate that $\cup_{R}$ and $\cap_{R}$ of $\Omega_{I}$-sets are not always $\Omega_{I}$-sets.

Example 3.19 Let $\left(\Gamma_{1},A\right)=\langle \frac{{\overset{˜}{\sigma }}_{\Gamma_{1}}}{\sigma_{\Gamma_{1}}},\frac{{\overset{˜}{\varsigma }}_{\Gamma_{1}}}{\varsigma_{\Gamma_{1}}}\rangle$ and $\left(\Gamma_{2},B\right)=\langle \frac{{\overset{˜}{\sigma }}_{\Gamma_{2}}}{\sigma_{\Gamma_{2}}},\frac{{\overset{˜}{\varsigma }}_{\Gamma_{2}}}{\varsigma_{\Gamma_{2}}}\rangle \in \Omega_{\left(\Gamma ,Z\right)}$ be two $\Omega_{I}$-sets on the universal set X with ${\overset{˜}{\sigma }}_{\Gamma_{1}}=[0.1,0.25],\sigma_{\Gamma_{1}}=0.2;{\overset{˜}{\varsigma }}_{\Gamma_{1}}=[0.3,0.6],\varsigma_{\Gamma_{1}}=0.4$ and ${\overset{˜}{\sigma }}_{\Gamma_{2}}=[0.35,0.5],\sigma_{\Gamma_{2}}=0.45;{\overset{˜}{\varsigma }}_{\Gamma_{2}}=[0.15,0.3],\varsigma_{\Gamma_{2}}=0.2$. Now

1. Let $\left(\Gamma_{1},A\right)\cup_{R}\left(\Gamma_{2},B\right)=\left(\Gamma_{3},C\right)$ with ${\overset{˜}{\sigma }}_{\Gamma_{3}}=[0.35,0.5],\sigma_{\Gamma_{3}}=0.2;{\overset{˜}{\varsigma }}_{\Gamma_{3}}=[0.15,0.3],\varsigma_{\Gamma_{3}}=0.4$ which is not $\Omega_{I}$-set on X.
2. Let $\left(\Gamma_{1},A\right)\cap_{R}\left(\Gamma_{2},B\right)=\left(\Gamma_{4},C\right)$ with ${\overset{˜}{\sigma }}_{\Gamma_{4}}=[0.1,0.25],\sigma_{\Gamma_{4}}=0.45;{\overset{˜}{\varsigma }}_{\Gamma_{4}}=[0.3,0.6],\varsigma_{\Gamma_{4}}=0.2$ which is not $\Omega_{I}$-set on X.

The $\cup_{R}$ and $\cap_{R}$ of $\Omega_{E}$-sets may not be $\Omega_{E}$-sets, as we will demonstrate in the following example.

Example 3.20 Let $\left(\Gamma_{1},A\right)=\langle \frac{{\overset{˜}{\sigma }}_{\Gamma_{1}}}{\sigma_{\Gamma_{1}}},\frac{{\overset{˜}{\varsigma }}_{\Gamma_{1}}}{\varsigma_{\Gamma_{1}}}\rangle$ and $\left(\Gamma_{2},B\right)=\langle \frac{{\overset{˜}{\sigma }}_{\Gamma_{2}}}{\sigma_{\Gamma_{2}}},\frac{{\overset{˜}{\varsigma }}_{\Gamma_{2}}}{\varsigma_{\Gamma_{2}}}\rangle \in \Omega_{\left(\Gamma ,Z\right)}$ be two $\Omega_{E}$-sets on the universal set X with ${\overset{˜}{\sigma }}_{\Gamma_{1}}=[0.15,0.30],\sigma_{\Gamma_{1}}=0.55;{\overset{˜}{\varsigma }}_{\Gamma_{1}}=[0.4,0.6],\varsigma_{\Gamma_{1}}=0.25$ and ${\overset{˜}{\sigma }}_{\Gamma_{2}}=[0.5,0.65],\sigma_{\Gamma_{2}}=0.75;{\overset{˜}{\varsigma }}_{\Gamma_{2}}=[0.2,0.3],\varsigma_{\Gamma_{2}}=0.1$. Now

1. Let $\left(\Gamma_{1},A\right)\cup_{R}\left(\Gamma_{2},B\right)=\left(\Gamma_{3},C\right)$ with ${\overset{˜}{\sigma }}_{\Gamma_{3}}=[0.5,0.65],\sigma_{\Gamma_{3}}=0.55;{\overset{˜}{\varsigma }}_{\Gamma_{3}}=[0.2,0.3],\varsigma_{\Gamma_{3}}=0.25$ which is not $\Omega_{E}$-set on X.
2. Let $\left(\Gamma_{1},A\right)\cap_{R}\left(\Gamma_{2},B\right)=\left(\Gamma_{4},C\right)$ with ${\overset{˜}{\sigma }}_{\Gamma_{4}}=[0.15,0.3],\sigma_{\Gamma_{4}}=0.75;{\overset{˜}{\varsigma }}_{\Gamma_{4}}=[0.4,0.6],\varsigma_{\Gamma_{4}}=0.1$ which is not $\Omega_{E}$-set on X.

We have shown in Example 3.19 that the $\cup_{R}$ of two $\Omega_{I}$-sets is not necessarily an $\Omega_{I}$-set. The following theorem is meant to present a condition for the $\cup_{R}$ of two $\Omega_{I}$-sets to be an $\Omega_{I}$-set.

Theorem 3.21 *Let*$\left(\Gamma_{1},A\right)=\langle \frac{{\overset{˜}{\sigma }}_{\Gamma_{1}}}{\sigma_{\Gamma_{1}}},\frac{{\overset{˜}{\varsigma }}_{\Gamma_{1}}}{\varsigma_{\Gamma_{1}}}\rangle$*and*$\left(\Gamma_{2},B\right)=\langle \frac{{\overset{˜}{\sigma }}_{\Gamma_{2}}}{\sigma_{\Gamma_{2}}},\frac{{\overset{˜}{\varsigma }}_{\Gamma_{2}}}{\varsigma_{\Gamma_{2}}}\rangle$*be any two*$\Omega_{I}$*-sets on the universal set*X*such that*$\text{max}\{\sigma_{\Gamma_{1}}^{L},\sigma_{\Gamma_{2}}^{L}\}\leq \text{min}\{\sigma_{\Gamma_{1}},\sigma_{\Gamma_{2}}\}$*and*$\text{min}\{\varsigma_{\Gamma_{1}}^{U},\varsigma_{\Gamma_{2}}^{U}\}\geq \text{max}\{\varsigma_{\Gamma_{1}},\varsigma_{\Gamma_{2}}\}$*, then*$\cup_{R}$*of*$\left(\Gamma_{1},A\right)$*and*$\left(\Gamma_{2},B\right)$*is*$\Omega_{I}$*-set on*X*.*

Proof Since $\left(\Gamma_{1},A\right)$ and $\left(\Gamma_{2},B\right)$ are $\Omega_{I}$-sets. So,

$$\sigma_{\Gamma_{1}}^{L}\leq \sigma_{\Gamma_{1}}\leq \sigma_{\Gamma_{1}}^{U}\;\;\;\text{and }\;\;\;\varsigma_{\Gamma_{1}}^{L}\leq \varsigma_{\Gamma_{1}}\leq \varsigma_{\Gamma_{1}}^{U}\;\sigma_{\Gamma_{2}}^{L}\leq \sigma_{\Gamma_{2}}\leq \sigma_{\Gamma_{2}}^{U}\;\;\;\text{and }\;\;\;\varsigma_{\Gamma_{2}}^{L}\leq \varsigma_{\Gamma_{2}}\leq \varsigma_{\Gamma_{2}}^{U}\Rightarrow \text{min}\{\sigma_{\Gamma_{1}},\sigma_{\Gamma_{2}}\}\leq \text{max}\{\sigma_{\Gamma_{1}}^{U},\sigma_{\Gamma_{2}}^{U}\}\;\;\;\text{and }\;\;\;\text{max}\{\varsigma_{\Gamma_{1}},\varsigma_{\Gamma_{2}}\}\geq \text{min}\{\varsigma_{\Gamma_{1}}^{L},\varsigma_{\Gamma_{2}}^{L}\}.$$

By given conditions it follows that

$$\text{max}\{\sigma_{\Gamma_{1}}^{L},\sigma_{\Gamma_{2}}^{L}\}\leq \text{min}\{\sigma_{\Gamma_{1}},\sigma_{\Gamma_{2}}\}\;\;\;\text{and }\;\;\;\text{min}\{\sigma_{\Gamma_{1}}^{U},\sigma_{\Gamma_{2}}^{U}\}\geq \text{max}\{\varsigma_{\Gamma_{1}},\varsigma_{\Gamma_{2}}\}\leq \text{max}\{\sigma_{\Gamma_{1}}^{U},\sigma_{\Gamma_{2}}^{U}\}\;\;\;\text{and }\;\;\;\geq \text{min}\{\varsigma_{\Gamma_{1}}^{L},\varsigma_{\Gamma_{2}}^{L}\}\Rightarrow \text{min}\{\sigma_{\Gamma_{1}},\sigma_{\Gamma_{2}}\}\in \text{rmax}\{{\overset{˜}{\sigma }}_{\Gamma_{1}},{\overset{˜}{\sigma }}_{\Gamma_{2}}\}\;\;\;\text{and }\;\;\;\text{max}\{\varsigma_{\Gamma_{1}},\varsigma_{\Gamma_{2}}\}\in \text{rmin}\{{\overset{˜}{\varsigma }}_{\Gamma_{1}},{\overset{˜}{\varsigma }}_{\Gamma_{2}}\}.$$

By Definition 3.12 $\cup_{R}$ of $\left(\Gamma_{1},A\right)$ and $\left(\Gamma_{2},B\right)$ is $\Omega_{I}$-set on X. □

In this theorem, $\cup_{R}$ of two $\Omega_{I}$-sets is again a $\Omega_{I}$-set under the following two constraints:

1. The maximum value of lower limits of the membership interval of both sets is less than or equal to the minimum membership value of both sets.
2. The minimum value of the upper limits of the non-membership interval of both sets is greater than or equal to the maximum non-membership value of both sets.

Theorem 3.22 *Let*$\left(\Gamma_{1},A\right)=\langle \frac{{\overset{˜}{\sigma }}_{\Gamma_{1}}}{\sigma_{\Gamma_{1}}},\frac{{\overset{˜}{\varsigma }}_{\Gamma_{1}}}{\varsigma_{\Gamma_{1}}}\rangle$*and*$\left(\Gamma_{2},B\right)=\langle \frac{{\overset{˜}{\sigma }}_{\Gamma_{2}}}{\sigma_{\Gamma_{2}}},\frac{{\overset{˜}{\varsigma }}_{\Gamma_{2}}}{\varsigma_{\Gamma_{2}}}\rangle$*be any two*$\Omega_{I}$*-sets on the universal set*X*such that*$\text{min}\{\sigma_{\Gamma_{1}}^{U},\sigma_{\Gamma_{2}}^{U}\}\geq \text{max}\{\sigma_{\Gamma_{1}},\sigma_{\Gamma_{2}}\}$*and*$\text{max}\{\varsigma_{\Gamma_{1}}^{L},\varsigma_{\Gamma_{2}}^{L}\}\leq \text{min}\{\varsigma_{\Gamma_{1}},\varsigma_{\Gamma_{2}}\}$*, then*$\cap_{R}$*of*$\left(\Gamma_{1},A\right)$*and*$\left(\Gamma_{2},B\right)$*is*$\Omega_{I}$*-set on*X*.*

Proof Since $\left(\Gamma_{1},A\right)$ and $\left(\Gamma_{2},B\right)$ are $\Omega_{I}$-sets. So,

$$\sigma_{\Gamma_{1}}^{L}\leq \sigma_{\Gamma_{1}}\leq \sigma_{\Gamma_{1}}^{U}\;\;\;\text{and }\;\;\;\varsigma_{\Gamma_{1}}^{L}\leq \varsigma_{\Gamma_{1}}\leq \varsigma_{\Gamma_{1}}^{U}\;\sigma_{\Gamma_{2}}^{L}\leq \sigma_{\Gamma_{2}}\leq \sigma_{\Gamma_{2}}^{U}\;\;\;\text{and }\;\;\;\varsigma_{\Gamma_{2}}^{L}\leq \varsigma_{\Gamma_{2}}\leq \varsigma_{\Gamma_{2}}^{U}\Rightarrow \text{max}\{\sigma_{\Gamma_{1}},\sigma_{\Gamma_{2}}\}\geq \text{min}\{\sigma_{\Gamma_{1}}^{L},\sigma_{\Gamma_{2}}^{L}\}\;\;\;\text{and }\;\;\;\text{min}\{\varsigma_{\Gamma_{1}},\varsigma_{\Gamma_{2}}\}\leq \text{max}\{\varsigma_{\Gamma_{1}}^{U},\varsigma_{\Gamma_{2}}^{U}\}.$$

By given conditions it follows that

$$\text{min}\{\sigma_{\Gamma_{1}}^{U},\sigma_{\Gamma_{2}}^{U}\}\geq \text{max}\{\sigma_{\Gamma_{1}},\sigma_{\Gamma_{2}}\}\;\;\;\text{and }\;\;\;\text{max}\{\sigma_{\Gamma_{1}}^{L},\sigma_{\Gamma_{2}}^{L}\}\leq \text{min}\{\varsigma_{\Gamma_{1}},\varsigma_{\Gamma_{2}}\}\geq \text{min}\{\sigma_{\Gamma_{1}}^{L},\sigma_{\Gamma_{2}}^{L}\}\;\;\;\text{and }\;\;\;\leq \text{max}\{\varsigma_{\Gamma_{1}}^{U},\varsigma_{\Gamma_{2}}^{U}\}\Rightarrow \text{max}\{\sigma_{\Gamma_{1}},\sigma_{\Gamma_{2}}\}\in \text{rmin}\{{\overset{˜}{\sigma }}_{\Gamma_{1}},{\overset{˜}{\sigma }}_{\Gamma_{2}}\}\;\;\;\text{and }\;\;\;\text{min}\{\varsigma_{\Gamma_{1}},\varsigma_{\Gamma_{2}}\}\in \text{rmax}\{{\overset{˜}{\varsigma }}_{\Gamma_{1}},{\overset{˜}{\varsigma }}_{\Gamma_{2}}\}.$$

By Definition 3.13 $\cap_{R}$ of $\left(\Gamma_{1},A\right)$ and $\left(\Gamma_{2},B\right)$ is $\Omega_{I}$-set on X. □

In this theorem, $\cap_{R}$ of two $\Omega_{I}$-sets is again a $\Omega_{I}$-set under the following two constraints:

1. The minimum value of the upper limits of the membership interval of both sets is greater than or equal to the maximum membership value of both sets.
2. The maximum value of the lower limits of the non-membership interval of both sets is less than or equal to the minimum non-membership value of both sets.

If we swap out $\sigma_{\Gamma_{1}}$ for $\sigma_{\Gamma_{2}}$ and $\varsigma_{\Gamma_{1}}$ for $\varsigma_{\Gamma_{2}}$ for two Ω-sets $\left(\Gamma_{1},A\right)=\langle \frac{{\overset{˜}{\sigma }}_{\Gamma_{1}}}{\sigma_{\Gamma_{1}}},\frac{{\overset{˜}{\varsigma }}_{\Gamma_{1}}}{\varsigma_{\Gamma_{1}}}\rangle$ and $\left(\Gamma_{2},B\right)=\langle \frac{{\overset{˜}{\sigma }}_{\Gamma_{2}}}{\sigma_{\Gamma_{2}}},\frac{{\overset{˜}{\varsigma }}_{\Gamma_{2}}}{\varsigma_{\Gamma_{2}}}\rangle$ on the universal set X. These Ω-sets on X are denoted by ${\left(\Gamma_{1},A\right)}^{⁎}=\langle \frac{{\overset{˜}{\sigma }}_{\Gamma_{1}}}{\sigma_{\Gamma_{2}}},\frac{{\overset{˜}{\varsigma }}_{\Gamma_{1}}}{\varsigma_{\Gamma_{2}}}\rangle$ and ${\left(\Gamma_{2},B\right)}^{⁎}=\langle \frac{{\overset{˜}{\sigma }}_{\Gamma_{2}}}{\sigma_{\Gamma_{1}}},\frac{{\overset{˜}{\varsigma }}_{\Gamma_{2}}}{\varsigma_{\Gamma_{1}}}\rangle$ respectively.

The following example shows that ${\left(\Gamma_{1},A\right)}^{⁎}$ and ${\left(\Gamma_{2},B\right)}^{⁎}$ do not have to be $\Omega_{I}$-sets on X for any two $\Omega_{E}$-sets $\left(\Gamma_{1},A\right)$ and $\left(\Gamma_{2},B\right)$ on X.

Example 3.23 Let $\left(\Gamma_{1},A\right)=\langle \frac{{\overset{˜}{\sigma }}_{\Gamma_{1}}}{\sigma_{\Gamma_{1}}},\frac{{\overset{˜}{\varsigma }}_{\Gamma_{1}}}{\varsigma_{\Gamma_{1}}}\rangle$ and $\left(\Gamma_{2},B\right)=\langle \frac{{\overset{˜}{\sigma }}_{\Gamma_{2}}}{\sigma_{\Gamma_{2}}},\frac{{\overset{˜}{\varsigma }}_{\Gamma_{2}}}{\varsigma_{\Gamma_{2}}}\rangle$ be any two $\Omega_{E}$-sets on the universal set $X=\{C_{1},C_{2}\}$ defined in Table 3 for all $\vartheta_{j}\in A,B$. Clearly ${\left(\Gamma_{1},A\right)}^{⁎}$ and ${\left(\Gamma_{2},B\right)}^{⁎}$ are not $\Omega_{E}$-sets on X.

**Table 3:** Tabular form of $\left(\Gamma_{1},A\right)\cup_{P}\left(\Gamma_{2},B\right)$ and $\left(\Gamma_{1},A\right)\cap_{P}\left(\Gamma_{2},B\right)\forall \vartheta_{j}\in A\cap B$.

X	$\left(\Gamma_{1},A\right)$	$\left(\Gamma_{2},B\right)$	$\left(\Gamma_{1},A\right)\cup_{P}\left(\Gamma_{2},B\right)$	$\left(\Gamma_{1},A\right)\cap_{P}\left(\Gamma_{2},B\right)$
$C_{1}$	$\langle \frac{\left[0.45,0.6\right]}{0.65},\frac{\left[0.1,0.25\right]}{0.3}\rangle$	$\langle \frac{\left[0.1,0.35\right]}{0.4},\frac{\left[0.4,0.55\right]}{0.35}\rangle$	$\langle \frac{\left[0.45,0.6\right]}{0.65},\frac{\left[0.1,0.25\right]}{0.3}\rangle$	$\langle \frac{\left[0.1,0.35\right]}{0.4},\frac{\left[0.4,0.55\right]}{0.35}\rangle$
$C_{2}$	$\langle \frac{\left[0.2,0.35\right]}{0.15},\frac{\left[0.4,0.65\right]}{0.7}\rangle$	$\langle \frac{\left[0.3,0.45\right]}{0.5},\frac{\left[0.15,0.3\right]}{0.1}\rangle$	$\langle \frac{\left[0.3,0.45\right]}{0.5},\frac{\left[0.15,0.3\right]}{0.1}\rangle$	$\langle \frac{\left[0.2,0.35\right]}{0.15},\frac{\left[0.4,0.65\right]}{0.7}\rangle$

We are going to demonstrate that the $\cup_{P}$ of two $\Omega_{E}$-set in X may not be an $\Omega_{I}$-set with the example that follows as.

Example 3.24 Examine two $\Omega_{E}$-set $\left(\Gamma_{1},A\right)$ and $\left(\Gamma_{2},B\right)$ in X, given in Table 3. In this case $\left(\Gamma_{1},A\right)\cup_{P}\left(\Gamma_{2},B\right)$ and $\left(\Gamma_{1},A\right)\cap_{P}\left(\Gamma_{2},B\right)$ are not $\Omega_{I}$-sets on the universal set X as shown in Table 4.

**Table 4:** Tabular form of $\left(\Gamma_{1},A\right)\cup_{R}\left(\Gamma_{2},B\right),\;\forall \;\vartheta_{j}\in A\cap B$.

X	$\left(\Gamma_{1},A\right)$	$\left(\Gamma_{2},B\right)$	$\left(\Gamma_{1},A\right)\cup_{R}\left(\Gamma_{2},B\right)$
$C_{1}$	$\langle \frac{\left[0.2,0.3\right]}{0.5},\frac{\left[0.4,0.5\right]}{0.25}\rangle$	$\langle \frac{\left[0.4,0.6\right]}{0.7},\frac{\left[0.2,0.4\right]}{0.2}\rangle$	$\langle \frac{\left[0.4,0.6\right]}{0.5},\frac{\left[0.2,0.4\right]}{0.25}\rangle$
$C_{2}$	$\langle \frac{\left[0.1,0.3\right]}{0.6},\frac{\left[0.5,0.7\right]}{0.3}\rangle$	$\langle \frac{\left[0.4,0.6\right]}{0.5},\frac{\left[0.2,0.4\right]}{0.1}\rangle$	$\langle \frac{\left[0.4,0.6\right]}{0.5},\frac{\left[0.2,0.4\right]}{0.3}\rangle$

A criterion for the $\cup_{P}$ of two $\Omega_{E}$-sets to be an $\Omega_{I}$-set is identified in the following result.

Theorem 3.25 *For two*$\Omega_{E}$*-sets*$\left(\Gamma_{1},A\right)=\langle \frac{{\overset{˜}{\sigma }}_{\Gamma_{1}}}{\sigma_{\Gamma_{1}}},\frac{{\overset{˜}{\varsigma }}_{\Gamma_{1}}}{\varsigma_{\Gamma_{1}}}\rangle$*and*$\left(\Gamma_{2},B\right)=\langle \frac{{\overset{˜}{\sigma }}_{\Gamma_{2}}}{\sigma_{\Gamma_{2}}},\frac{{\overset{˜}{\varsigma }}_{\Gamma_{2}}}{\varsigma_{\Gamma_{2}}}\rangle$*on the universal set*X*. If*${\left(\Gamma_{1},A\right)}^{⁎}=\langle \frac{{\overset{˜}{\sigma }}_{\Gamma_{1}}}{\sigma_{\Gamma_{2}}},\frac{{\overset{˜}{\varsigma }}_{\Gamma_{1}}}{\varsigma_{\Gamma_{2}}}\rangle$*and*${\left(\Gamma_{2},B\right)}^{⁎}=\langle \frac{{\overset{˜}{\sigma }}_{\Gamma_{2}}}{\sigma_{\Gamma_{1}}},\frac{{\overset{˜}{\varsigma }}_{\Gamma_{2}}}{\varsigma_{\Gamma_{1}}}\rangle$*are*$\Omega_{I}$*-sets on*X*. Then*$\left(\Gamma_{1},A\right)\cup_{P}\left(\Gamma_{2},B\right)$*and*$\left(\Gamma_{1},A\right)\cap_{P}\left(\Gamma_{2},B\right)$*are*$\Omega_{I}$*-sets on*X*.*

Proof Since $\left(\Gamma_{1},A\right)$ and $\left(\Gamma_{2},B\right)$ are $\Omega_{E}$-sets on X. So,

$$\sigma_{\Gamma_{1}}\notin (\sigma_{\Gamma_{1}}^{L},\sigma_{\Gamma_{1}}^{U})\;\;\text{and }\;\;\varsigma_{\Gamma_{1}}\notin (\varsigma_{\Gamma_{1}}^{L},\varsigma_{\Gamma_{1}}^{U}),\sigma_{\Gamma_{2}}\notin (\sigma_{\Gamma_{2}}^{L},\sigma_{\Gamma_{2}}^{U})\;\;\text{and }\;\;\varsigma_{\Gamma_{2}}\notin (\varsigma_{\Gamma_{2}}^{L},\varsigma_{\Gamma_{2}}^{U}),$$

for all $x\in X,\vartheta_{j}\in Z$. Also ${\left(\Gamma_{1},A\right)}^{⁎}$ and ${\left(\Gamma_{2},B\right)}^{⁎}$ are $\Omega_{I}$-sets on X. So,

$$\sigma_{\Gamma_{1}}^{L}\leq \sigma_{\Gamma_{2}}\leq \sigma_{\Gamma_{1}}^{U}\;\;\text{and }\;\;\varsigma_{\Gamma_{1}}^{L}\leq \varsigma_{\Gamma_{2}}\leq \varsigma_{\Gamma_{1}}^{U},\sigma_{\Gamma_{2}}^{L}\leq \sigma_{\Gamma_{1}}\leq \sigma_{\Gamma_{2}}^{U}\;\;\text{and }\;\;\varsigma_{\Gamma_{2}}^{L}\leq \varsigma_{\Gamma_{1}}\leq \varsigma_{\Gamma_{2}}^{U},$$

for all $x\in X,\vartheta_{j}\in Z$. We have the following cases for any x∈X

$$\text{Case 1}\;\sigma_{\Gamma_{1}}\leq \sigma_{\Gamma_{1}}^{L}\leq \sigma_{\Gamma_{2}}\leq \sigma_{\Gamma_{1}}^{U}\;\;\;\;\;\;\;\;\;;\;\varsigma_{\Gamma_{1}}\leq \varsigma_{\Gamma_{1}}^{L}\leq \varsigma_{\Gamma_{2}}\leq \varsigma_{\Gamma_{1}}^{U}\;\;\;\sigma_{\Gamma_{2}}\leq \sigma_{\Gamma_{2}}^{L}\leq \sigma_{\Gamma_{1}}\leq \sigma_{\Gamma_{2}}^{U}\;\text{and }\;\varsigma_{\Gamma_{2}}\leq \varsigma_{\Gamma_{2}}^{L}\leq \varsigma_{\Gamma_{1}}\leq \varsigma_{\Gamma_{2}}^{U}.\;\;\text{Case 2}\;\sigma_{\Gamma_{1}}^{L}\leq \sigma_{\Gamma_{2}}\leq \sigma_{\Gamma_{1}}^{U}\leq \sigma_{\Gamma_{1}}\;\;\;\;\;\;\;\;\;;\;\varsigma_{\Gamma_{1}}^{L}\leq \varsigma_{\Gamma_{2}}\leq \varsigma_{\Gamma_{1}}^{U}\leq \varsigma_{\Gamma_{1}}\;\;\;\sigma_{\Gamma_{2}}^{L}\leq \sigma_{\Gamma_{1}}\leq \sigma_{\Gamma_{2}}^{U}\leq \sigma_{\Gamma_{2}}\;\text{and }\;\varsigma_{\Gamma_{2}}^{L}\leq \varsigma_{\Gamma_{1}}\leq \varsigma_{\Gamma_{2}}^{U}\leq \varsigma_{\Gamma_{2}}.\;\;\text{Case 3}\;\sigma_{\Gamma_{1}}\leq \sigma_{\Gamma_{1}}^{L}\leq \sigma_{\Gamma_{2}}\leq \sigma_{\Gamma_{1}}^{U}\;\;\;\;\;\;\;\;\;;\;\varsigma_{\Gamma_{1}}\leq \varsigma_{\Gamma_{1}}^{L}\leq \varsigma_{\Gamma_{2}}\leq \varsigma_{\Gamma_{1}}^{U}\;\;\;\sigma_{\Gamma_{2}}^{L}\leq \sigma_{\Gamma_{1}}\leq \sigma_{\Gamma_{2}}^{U}\leq \sigma_{\Gamma_{2}}\;\text{and }\;\varsigma_{\Gamma_{2}}^{L}\leq \varsigma_{\Gamma_{1}}\leq \varsigma_{\Gamma_{2}}^{U}\leq \varsigma_{\Gamma_{2}}.\;\;\text{Case 4}\;\sigma_{\Gamma_{1}}^{L}\leq \sigma_{\Gamma_{2}}\leq \sigma_{\Gamma_{1}}^{U}\leq \sigma_{\Gamma_{1}}\;\;\;\;\;\;\;\;\;;\;\varsigma_{\Gamma_{1}}^{L}\leq \varsigma_{\Gamma_{2}}\leq \varsigma_{\Gamma_{1}}^{U}\leq \varsigma_{\Gamma_{1}}\;\;\;\sigma_{\Gamma_{2}}\leq \sigma_{\Gamma_{2}}^{L}\leq \sigma_{\Gamma_{1}}\leq \sigma_{\Gamma_{2}}^{U}\;\text{and }\;\varsigma_{\Gamma_{2}}\leq \varsigma_{\Gamma_{2}}^{L}\leq \varsigma_{\Gamma_{1}}\leq \varsigma_{\Gamma_{2}}^{U}.$$

Since the explanations in every case are similar, we only take the first one into consideration. We have $\sigma_{\Gamma_{1}}=\sigma_{\Gamma_{1}}^{L}=\sigma_{\Gamma_{2}}=\sigma_{\Gamma_{2}}^{L}$ and $\varsigma_{\Gamma_{1}}=\varsigma_{\Gamma_{1}}^{L}=\varsigma_{\Gamma_{2}}=\varsigma_{\Gamma_{2}}^{L}$. Also ${\left(\Gamma_{1},A\right)}^{⁎}$ and ${\left(\Gamma_{2},B\right)}^{⁎}$ are $\Omega_{I}$-sets. So, $\sigma_{\Gamma_{2}}\leq \sigma_{\Gamma_{1}}^{U}$; $\varsigma_{\Gamma_{1}}^{L}\leq \varsigma_{\Gamma_{2}}$ and $\sigma_{\Gamma_{1}}\leq \sigma_{\Gamma_{2}}^{U}$; $\varsigma_{\Gamma_{2}}^{L}\leq \varsigma_{\Gamma_{1}}$. It follows that:

$${\left(\sigma_{\Gamma_{1}}\cup \sigma_{\Gamma_{2}}\right)}^{L}=\text{max}\{\sigma_{\Gamma_{1}}^{L},\sigma_{\Gamma_{2}}^{L}\}=\text{max}\{\sigma_{\Gamma_{1}},\sigma_{\Gamma_{2}}\}=\sigma_{\Gamma_{1}}\vee \sigma_{\Gamma_{2}}\leq \text{max}\{\sigma_{\Gamma_{1}}^{U},\sigma_{\Gamma_{2}}^{U}\}={\left(\sigma_{\Gamma_{1}}\cup \sigma_{\Gamma_{2}}\right)}^{U}\Rightarrow \;\;\;\;\;\text{max}\{\sigma_{\Gamma_{1}},\sigma_{\Gamma_{2}}\}\in \text{rmax}\{{\overset{˜}{\sigma }}_{\Gamma_{1}},{\overset{˜}{\sigma }}_{\Gamma_{2}}\},\text{and}{\left(\varsigma_{\Gamma_{1}}\cap \varsigma_{\Gamma_{2}}\right)}^{U}=\text{min}\{\varsigma_{\Gamma_{1}}^{U},\varsigma_{\Gamma_{2}}^{U}\}=\text{min}\{\varsigma_{\Gamma_{1}},\varsigma_{\Gamma_{2}}\}=\varsigma_{\Gamma_{1}}\wedge \varsigma_{\Gamma_{2}}\geq \text{min}\{\varsigma_{\Gamma_{1}}^{L},\varsigma_{\Gamma_{2}}^{L}\}={\left(\varsigma_{\Gamma_{1}}\cap \varsigma_{\Gamma_{2}}\right)}^{L}\Rightarrow \;\;\;\;\;\text{min}\{\varsigma_{\Gamma_{1}},\varsigma_{\Gamma_{2}}\}\in \text{rmin}\{{\overset{˜}{\varsigma }}_{\Gamma_{1}},{\overset{˜}{\varsigma }}_{\Gamma_{2}}\}.$$

Hence $\left(\Gamma_{1},A\right)\cup_{P}\left(\Gamma_{2},B\right)$ is an $\Omega_{I}$-set on X. Similarly, $\left(\Gamma_{1},A\right)\cap_{P}\left(\Gamma_{2},B\right)$ can be performed. □

From the Example 3.24 it is clear that $\cup_{P}$ and $\cap_{P}$ of $\Omega_{E}$-sets are not necessarily $\Omega_{E}$-set on X. Next condition shows that the $\cup_{P}$ of two $\Omega_{E}$-sets is $\Omega_{E}$-set on X.

Theorem 3.26 *Let*$\left(\Gamma_{1},A\right)=\langle \frac{{\overset{˜}{\sigma }}_{\Gamma_{1}}}{\sigma_{\Gamma_{1}}},\frac{{\overset{˜}{\varsigma }}_{\Gamma_{1}}}{\varsigma_{\Gamma_{1}}}\rangle$*and*$\left(\Gamma_{2},B\right)=\langle \frac{{\overset{˜}{\sigma }}_{\Gamma_{2}}}{\sigma_{\Gamma_{2}}},\frac{{\overset{˜}{\varsigma }}_{\Gamma_{2}}}{\varsigma_{\Gamma_{2}}}\rangle$*be two*$\Omega_{E}$*-sets on the universal set*X*. If*${\left(\Gamma_{1},A\right)}^{⁎}=\langle \frac{{\overset{˜}{\sigma }}_{\Gamma_{1}}}{\sigma_{\Gamma_{2}}},\frac{{\overset{˜}{\varsigma }}_{\Gamma_{1}}}{\varsigma_{\Gamma_{2}}}\rangle$*and*${\left(\Gamma_{2},B\right)}^{⁎}=\langle \frac{{\overset{˜}{\sigma }}_{\Gamma_{2}}}{\sigma_{\Gamma_{1}}},\frac{{\overset{˜}{\varsigma }}_{\Gamma_{2}}}{\varsigma_{\Gamma_{1}}}\rangle$*are*$\Omega_{E}$*-sets on*X*. Then*$\left(\Gamma_{1},A\right)\cup_{P}\left(\Gamma_{2},B\right)$*is also*$\Omega_{E}$*-set on*X*.*

Proof Since $\left(\Gamma_{1},A\right),\left(\Gamma_{2},B\right),{\left(\Gamma_{1},A\right)}^{⁎}$ and ${\left(\Gamma_{2},B\right)}^{⁎}$ are $\Omega_{E}$-sets on X. So,

$$\sigma_{\Gamma_{1}}\notin (\sigma_{\Gamma_{1}}^{L},\sigma_{\Gamma_{1}}^{U})\;\;\text{and }\;\;\varsigma_{\Gamma_{1}}\notin (\varsigma_{\Gamma_{1}}^{L},\varsigma_{\Gamma_{1}}^{U}).\sigma_{\Gamma_{2}}\notin (\sigma_{\Gamma_{2}}^{L},\sigma_{\Gamma_{2}}^{U})\;\;\text{and }\;\;\varsigma_{\Gamma_{2}}\notin (\varsigma_{\Gamma_{2}}^{L},\varsigma_{\Gamma_{2}}^{U}).\sigma_{\Gamma_{2}}\notin (\sigma_{\Gamma_{1}}^{L},\sigma_{\Gamma_{1}}^{U})\;\;\text{and }\;\;\varsigma_{\Gamma_{2}}\notin (\varsigma_{\Gamma_{1}}^{L},\varsigma_{\Gamma_{1}}^{U}).\sigma_{\Gamma_{1}}\notin (\sigma_{\Gamma_{2}}^{L},\sigma_{\Gamma_{2}}^{U})\;\;\text{and }\;\;\varsigma_{\Gamma_{1}}\notin (\varsigma_{\Gamma_{2}}^{L},\varsigma_{\Gamma_{2}}^{U}).\text{Hence}\;\;\;\;\;\;\;\;\sigma_{\Gamma_{1}}\vee \sigma_{\Gamma_{2}}\notin (\text{max}\{\sigma_{\Gamma_{1}}^{L},\sigma_{\Gamma_{2}}^{L}\},\text{max}\{\sigma_{\Gamma_{1}}^{U},\sigma_{\Gamma_{2}}^{U}\})\Rightarrow \text{max}\{\sigma_{\Gamma_{1}},\sigma_{\Gamma_{2}}\}\notin \text{rmax}\{{\overset{˜}{\sigma }}_{\Gamma_{1}},{\overset{˜}{\sigma }}_{\Gamma_{2}}\}\text{and}\;\;\;\;\;\;\;\;\sigma_{\Gamma_{1}}\wedge \sigma_{\Gamma_{2}}\notin (\text{min}\{\sigma_{\Gamma_{1}}^{L},\sigma_{\Gamma_{2}}^{L}\},\text{min}\{\sigma_{\Gamma_{1}}^{U},\sigma_{\Gamma_{2}}^{U}\})\Rightarrow \text{min}\{\sigma_{\Gamma_{1}},\sigma_{\Gamma_{2}}\}\notin \text{rmin}\{{\overset{˜}{\sigma }}_{\Gamma_{1}},{\overset{˜}{\sigma }}_{\Gamma_{2}}\}$$

Therefore $\left(\Gamma_{1},A\right)\cup_{P}\left(\Gamma_{2},B\right)$ is $\Omega_{E}$-set on X. □

Note that $\cap_{P}$ of two $\Omega_{E}$-sets may not be an $\Omega_{E}$-set. Now, here we provide a condition for the $\cap_{P}$ of two $\Omega_{E}$-sets to be an $\Omega_{E}$-set on X.

Theorem 3.27 *Let*$\left(\Gamma_{1},A\right)=\langle \frac{{\overset{˜}{\sigma }}_{\Gamma_{1}}}{\sigma_{\Gamma_{1}}},\frac{{\overset{˜}{\varsigma }}_{\Gamma_{1}}}{\varsigma_{\Gamma_{1}}}\rangle$*and*$\left(\Gamma_{2},B\right)=\langle \frac{{\overset{˜}{\sigma }}_{\Gamma_{2}}}{\sigma_{\Gamma_{2}}},\frac{{\overset{˜}{\varsigma }}_{\Gamma_{2}}}{\varsigma_{\Gamma_{2}}}\rangle$*be two*$\Omega_{E}$*-sets on the universal set*X*such that*

$$\text{min}\{\text{max}\{\sigma_{\Gamma_{1}}^{L},\sigma_{\Gamma_{2}}^{U}\},\text{max}\{\sigma_{\Gamma_{1}}^{U},\sigma_{\Gamma_{2}}^{L}\}\}\geq \;(\sigma_{\Gamma_{1}}\wedge \sigma_{\Gamma_{2}})\;>\text{max}\{\text{min}\{\sigma_{\Gamma_{1}}^{L},\sigma_{\Gamma_{2}}^{U}\},\text{min}\{\sigma_{\Gamma_{1}}^{U},\sigma_{\Gamma_{2}}^{L}\}\}\text{and}\;\;\;\text{min}\{\text{max}\{\varsigma_{\Gamma_{1}}^{L},\varsigma_{\Gamma_{2}}^{U}\},\text{max}\{\varsigma_{\Gamma_{1}}^{U},\varsigma_{\Gamma_{2}}^{L}\}\}>\;(\varsigma_{\Gamma_{1}}\vee \varsigma_{\Gamma_{2}})\;\geq \text{max}\{\text{min}\{\varsigma_{\Gamma_{1}}^{L},\varsigma_{\Gamma_{2}}^{U}\},\text{min}\{\varsigma_{\Gamma_{1}}^{U},\varsigma_{\Gamma_{2}}^{L}\}\}$$

*then*$\left(\Gamma_{1},A\right)\cap_{P}\left(\Gamma_{2},B\right)$*is an*$\Omega_{E}$*-set on*X*.*

Proof For each x∈X, we take

$$\alpha_{x}=\text{min}\{\text{max}\{\sigma_{\Gamma_{1}}^{L},\sigma_{\Gamma_{2}}^{U}\},\text{max}\{\sigma_{\Gamma_{1}}^{U},\sigma_{\Gamma_{2}}^{L}\}\},\beta_{x}=\text{max}\{\text{min}\{\sigma_{\Gamma_{1}}^{L},\sigma_{\Gamma_{2}}^{U}\},\text{min}\{\sigma_{\Gamma_{1}}^{U},\sigma_{\Gamma_{2}}^{L}\}\},\alpha_{x}^{⁎}=\text{min}\{\text{max}\{\varsigma_{\Gamma_{1}}^{L},\varsigma_{\Gamma_{2}}^{U}\},\text{max}\{\varsigma_{\Gamma_{1}}^{U},\varsigma_{\Gamma_{2}}^{L}\}\},\beta_{x}^{⁎}=\text{max}\{\text{min}\{\varsigma_{\Gamma_{1}}^{L},\varsigma_{\Gamma_{2}}^{U}\},\text{min}\{\varsigma_{\Gamma_{1}}^{U},\varsigma_{\Gamma_{2}}^{L}\}\}.$$

Now $\alpha_{x}$ is one of the $\sigma_{\Gamma_{1}}^{L},\sigma_{\Gamma_{1}}^{U},\sigma_{\Gamma_{2}}^{L},\sigma_{\Gamma_{2}}^{U}$ and $\alpha_{x}^{⁎}$ is one of the $\varsigma_{\Gamma_{1}}^{L},\varsigma_{\Gamma_{1}}^{U},\varsigma_{\Gamma_{2}}^{L},\varsigma_{\Gamma_{2}}^{U}$. With out loss of generality we will consider only when $\alpha_{x}=\sigma_{\Gamma_{1}}^{L}$ and $\alpha_{x}^{⁎}=\varsigma_{\Gamma_{1}}^{L}$ or $\alpha_{x}=\sigma_{\Gamma_{1}}^{U}$ and $\alpha_{x}^{⁎}=\varsigma_{\Gamma_{1}}^{U}$. For the all remaining cases, the arguments are similar to these cases.

If $\alpha_{x}=\sigma_{\Gamma_{1}}^{L}$ and $\alpha_{x}^{⁎}=\varsigma_{\Gamma_{1}}^{L}$, then $\sigma_{\Gamma_{2}}^{L}\leq \sigma_{\Gamma_{2}}^{U}\leq \sigma_{\Gamma_{1}}^{L}\leq \sigma_{\Gamma_{1}}^{U}$, $\varsigma_{\Gamma_{2}}^{L}\leq \varsigma_{\Gamma_{2}}^{U}\leq \varsigma_{\Gamma_{1}}^{L}\leq \varsigma_{\Gamma_{1}}^{U}$. So, $\beta_{x}=\sigma_{\Gamma_{2}}^{U}$ and $\beta_{x}^{⁎}=\varsigma_{\Gamma_{2}}^{U}$. Thus

$$\sigma_{\Gamma_{2}}^{L}=\text{min}\{\sigma_{\Gamma_{1}}^{L},\sigma_{\Gamma_{2}}^{L}\}\leq \text{min}\{\sigma_{\Gamma_{1}}^{U},\sigma_{\Gamma_{2}}^{U}\}=\varsigma_{\Gamma_{2}}^{U}=\beta_{x}^{⁎}<(\sigma_{\Gamma_{1}}\wedge \sigma_{\Gamma_{2}})\varsigma_{\Gamma_{1}}^{L}=\text{max}\{\varsigma_{\Gamma_{1}}^{L},\varsigma_{\Gamma_{2}}^{L}\}=\alpha_{x}^{⁎}>(\varsigma_{\Gamma_{1}}\vee \varsigma_{\Gamma_{2}})\text{So,}\;\;\;\;\;\;\;\;\;\;\;\;\;\;\;\;\;\;\;\;\;\;\;\;\;\;\;\;\;(\sigma_{\Gamma_{1}}\wedge \sigma_{\Gamma_{2}})\notin (\text{min}\{\sigma_{\Gamma_{1}}^{L},\sigma_{\Gamma_{2}}^{L}\},\text{min}\{\sigma_{\Gamma_{1}}^{U},\sigma_{\Gamma_{2}}^{U}\})=\text{rmin}\{{\overset{˜}{\sigma }}_{\Gamma_{1}},{\overset{˜}{\sigma }}_{\Gamma_{2}}\}\text{and}\;\;\;\;\;\;\;\;\;\;\;\;\;\;\;\;\;\;\;\;\;\;\;\;\;\;\;\;\;(\varsigma_{\Gamma_{1}}\vee \varsigma_{\Gamma_{2}})\notin (\text{max}\{\varsigma_{\Gamma_{1}}^{L},\varsigma_{\Gamma_{2}}^{L}\},\text{max}\{\varsigma_{\Gamma_{1}}^{U},\varsigma_{\Gamma_{2}}^{U}\})=\text{rmax}\{{\overset{˜}{\varsigma }}_{\Gamma_{1}},{\overset{˜}{\varsigma }}_{\Gamma_{2}}\}.$$

Thus, in this case $\left(\Gamma_{1},A\right)\cap_{P}\left(\Gamma_{2},B\right)$ is an $\Omega_{E}$-set on X.

If we take $\alpha_{x}=\sigma_{\Gamma_{1}}^{U}$ and $\alpha_{x}^{⁎}=\varsigma_{\Gamma_{1}}^{U}$, then

$$\sigma_{\Gamma_{2}}^{L}\leq \sigma_{\Gamma_{1}}^{U}\leq \sigma_{\Gamma_{2}}^{U}\;\;\;\;\;\;\;\;\text{and}\;\;\;\;\;\;\;\;\varsigma_{\Gamma_{2}}^{L}\leq \varsigma_{\Gamma_{1}}^{U}\leq \varsigma_{\Gamma_{2}}^{U}\;\text{so,}\;\;\;\;\;\;\beta_{x}=\text{max}\{\sigma_{\Gamma_{1}}^{L},\sigma_{\Gamma_{2}}^{L}\}\;\;\;\;\;\;\;\;\text{and}\;\;\;\;\;\;\;\;\beta_{x}^{⁎}=\text{max}\{\varsigma_{\Gamma_{1}}^{L},\varsigma_{\Gamma_{2}}^{L}\}.$$

Consider that $\beta_{x}=\sigma_{\Gamma_{1}}^{L}$ and $\beta_{x}^{⁎}=\varsigma_{\Gamma_{1}}^{L}$, then

$$\sigma_{\Gamma_{2}}^{L}\leq \sigma_{\Gamma_{1}}^{L}=\beta_{x}<(\sigma_{\Gamma_{1}}\wedge \sigma_{\Gamma_{2}})\leq \sigma_{\Gamma_{1}}^{U}\leq \sigma_{\Gamma_{2}}^{U}\;\;\;\;\;\;\;\text{and}\;\varsigma_{\Gamma_{2}}^{L}\leq \;\;\varsigma_{\Gamma_{1}}^{L}=\beta_{x}^{⁎}\leq (\varsigma_{\Gamma_{1}}\vee \varsigma_{\Gamma_{2}})<\varsigma_{\Gamma_{1}}^{U}\leq \varsigma_{\Gamma_{2}}^{U}.$$

Above inequalities arise following two cases.

$$\text{Case 1}\;\sigma_{\Gamma_{2}}^{L}\leq \sigma_{\Gamma_{1}}^{L}=\beta_{x}<(\sigma_{\Gamma_{1}}\wedge \sigma_{\Gamma_{2}})<\sigma_{\Gamma_{1}}^{U}\leq \sigma_{\Gamma_{2}}^{U}\;\;\;\;\;\;\;\text{and}\;\;\varsigma_{\Gamma_{2}}^{L}\leq \;\;\varsigma_{\Gamma_{1}}^{L}=\beta_{x}^{⁎}<(\varsigma_{\Gamma_{1}}\vee \varsigma_{\Gamma_{2}})<\varsigma_{\Gamma_{1}}^{U}\leq \varsigma_{\Gamma_{2}}^{U}\;\text{Case 2}\;\sigma_{\Gamma_{2}}^{L}\leq \sigma_{\Gamma_{1}}^{L}=\beta_{x}<(\sigma_{\Gamma_{1}}\wedge \sigma_{\Gamma_{2}})=\sigma_{\Gamma_{1}}^{U}\leq \sigma_{\Gamma_{2}}^{U}\;\;\;\;\;\;\;\text{and}\;\;\varsigma_{\Gamma_{2}}^{L}\leq \;\;\varsigma_{\Gamma_{1}}^{L}=\beta_{x}^{⁎}=(\varsigma_{\Gamma_{1}}\vee \varsigma_{\Gamma_{2}})<\varsigma_{\Gamma_{1}}^{U}\leq \varsigma_{\Gamma_{2}}^{U}.$$

Since $\left(\Gamma_{1},A\right)$ and $\left(\Gamma_{2},B\right)$ are $\Omega_{E}$-sets on X contradict the case 1. Case 2 implies that

$$(\sigma_{\Gamma_{1}}\wedge \sigma_{\Gamma_{2}})\notin (\text{min}\{\sigma_{\Gamma_{1}}^{L},\sigma_{\Gamma_{2}}^{L}\},\text{min}\{\sigma_{\Gamma_{1}}^{U},\sigma_{\Gamma_{2}}^{U}\})\;\;\;\;\;\;\;\;\;\;∵\;\;\;\;\;\;\;\text{min}\{\sigma_{\Gamma_{1}}^{U},\sigma_{\Gamma_{2}}^{U}\}=\sigma_{\Gamma_{1}}^{U}=(\sigma_{\Gamma_{1}}\wedge \sigma_{\Gamma_{2}})\text{and}\;\;\;(\varsigma_{\Gamma_{1}}\vee \varsigma_{\Gamma_{2}})\notin (\text{max}\{\varsigma_{\Gamma_{1}}^{L},\varsigma_{\Gamma_{2}}^{L}\},\text{max}\{\varsigma_{\Gamma_{1}}^{U},\varsigma_{\Gamma_{2}}^{U}\})\;\;\;\;\;\;\;\;\;\;∵\;\;\;\;\;\;\;\text{max}\{\varsigma_{\Gamma_{1}}^{L},\varsigma_{\Gamma_{2}}^{L}\}=\varsigma_{\Gamma_{1}}^{L}=(\varsigma_{\Gamma_{1}}\vee \varsigma_{\Gamma_{2}}).$$

Assume that $\beta_{x}=\sigma_{\Gamma_{2}}^{L}$ and $\beta_{x}^{⁎}=\varsigma_{\Gamma_{2}}^{L}$, then

$$\sigma_{\Gamma_{1}}^{L}\leq \;\;\sigma_{\Gamma_{2}}^{L}=\beta_{x}<(\sigma_{\Gamma_{1}}\wedge \sigma_{\Gamma_{2}})\leq \sigma_{\Gamma_{1}}^{U}\leq \sigma_{\Gamma_{2}}^{U}\;\;\;\;\;\;\;\text{and}\;\varsigma_{\Gamma_{1}}^{L}\leq \;\;\varsigma_{\Gamma_{2}}^{L}\;=\beta_{x}^{⁎}\leq (\varsigma_{\Gamma_{1}}\vee \varsigma_{\Gamma_{2}})\;\;<\varsigma_{\Gamma_{1}}^{U}\;\;\leq \varsigma_{\Gamma_{2}}^{U}.$$

We have following two cases.

$$\text{Case 1}\;\sigma_{\Gamma_{1}}^{L}\leq \sigma_{\Gamma_{2}}^{L}=\beta_{x}<(\sigma_{\Gamma_{1}}\wedge \sigma_{\Gamma_{2}})<\sigma_{\Gamma_{1}}^{U}\leq \sigma_{\Gamma_{2}}^{U}\;\;\;\;\;\;\;\text{and}\;\;\varsigma_{\Gamma_{1}}^{L}\leq \;\;\varsigma_{\Gamma_{2}}^{L}=\beta_{x}^{⁎}<(\varsigma_{\Gamma_{1}}\vee \varsigma_{\Gamma_{2}})\;\;<\varsigma_{\Gamma_{1}}^{U}\leq \varsigma_{\Gamma_{2}}^{U}\;\text{Case 2}\;\sigma_{\Gamma_{1}}^{L}\leq \sigma_{\Gamma_{2}}^{L}=\beta_{x}<(\sigma_{\Gamma_{1}}\wedge \sigma_{\Gamma_{2}})=\sigma_{\Gamma_{1}}^{U}\leq \sigma_{\Gamma_{2}}^{U}\;\;\;\;\;\;\;\text{and}\;\;\varsigma_{\Gamma_{1}}^{L}\leq \;\;\varsigma_{\Gamma_{2}}^{L}=\beta_{x}^{⁎}=(\varsigma_{\Gamma_{1}}\vee \varsigma_{\Gamma_{2}})\;\;<\varsigma_{\Gamma_{1}}^{U}\leq \varsigma_{\Gamma_{2}}^{U}.$$

Case 1 contradicts the fact that $\left(\Gamma_{1},A\right)$ and $\left(\Gamma_{2},B\right)$ are $\Omega_{E}$-sets on X. Case 2 implies that

$$(\sigma_{\Gamma_{1}}\wedge \sigma_{\Gamma_{2}})\notin (\text{min}\{\sigma_{\Gamma_{1}}^{L},\sigma_{\Gamma_{2}}^{L}\},\text{min}\{\sigma_{\Gamma_{1}}^{U},\sigma_{\Gamma_{2}}^{U}\})\;\;\;\;\;\;\;\;\;\;∵\;\;\;\;\;\;\;\text{min}\{\sigma_{\Gamma_{1}}^{U},\sigma_{\Gamma_{2}}^{U}\}=\sigma_{\Gamma_{1}}^{U}=(\sigma_{\Gamma_{1}}\wedge \sigma_{\Gamma_{2}})\text{and}\;\;\;(\varsigma_{\Gamma_{1}}\vee \varsigma_{\Gamma_{2}})\notin (\text{max}\{\varsigma_{\Gamma_{1}}^{L},\varsigma_{\Gamma_{2}}^{L}\},\text{max}\{\varsigma_{\Gamma_{1}}^{U},\varsigma_{\Gamma_{2}}^{U}\})\;\;\;\;\;\;\;\;\;\;∵\;\;\;\;\;\;\;\text{max}\{\varsigma_{\Gamma_{1}}^{L},\varsigma_{\Gamma_{2}}^{L}\}\;=\varsigma_{\Gamma_{2}}^{L}\;=(\varsigma_{\Gamma_{1}}\vee \varsigma_{\Gamma_{2}}).$$

We can obtain the similar result if we assume that $\beta_{x}=\sigma_{\Gamma_{2}}^{L},\beta_{x}^{⁎}=\varsigma_{\Gamma_{1}}^{L}\;\;\;\text{or}\;\;\;\beta_{x}=\sigma_{\Gamma_{1}}^{L},\beta_{x}^{⁎}=\varsigma_{\Gamma_{2}}^{L}\;\;\;\text{and}\;\;\;\beta_{x}=\sigma_{\Gamma_{2}}^{L},\beta_{x}^{⁎}=\varsigma_{\Gamma_{2}}^{L}$. Hence, $\left(\Gamma_{1},A\right)\cap_{P}\left(\Gamma_{2},B\right)$ is an $\Omega_{E}$-set on X. □

Example 3.28 Consider two $\Omega_{E}$-sets $\left(\Gamma_{1},A\right)$ and $\left(\Gamma_{2},B\right)$ in $X=\{C_{1},C_{2}\}$, defined in Table 5, Table 6 respectively. In this example $\left(\Gamma_{1},A\right)\cup_{R}\left(\Gamma_{2},B\right)$ and $\left(\Gamma_{1},A\right)\cap_{R}\left(\Gamma_{2},B\right)$ are not $\Omega_{E}$-sets on the universal set X as shown in Table 5, Table 6 respectively.

**Table 5:** Tabular form of $\left(\Gamma_{1},A\right)\cap_{R}\left(\Gamma_{2},B\right)\;\forall \;\vartheta_{j}\in A\cap B$.

X	$\left(\Gamma_{1},A\right)$	$\left(\Gamma_{2},B\right)$	$\left(\Gamma_{1},A\right)\cap_{R}\left(\Gamma_{2},B\right)$
$C_{1}$	$\langle \frac{\left[0.2,0.3\right]}{0.1},\frac{\left[0.4,0.5\right]}{0.55}\rangle$	$\langle \frac{\left[0.4,0.6\right]}{0.25},\frac{\left[0.25,0.4\right]}{0.45}\rangle$	$\langle \frac{\left[0.2,0.3\right]}{0.25},\frac{\left[0.4,0.5\right]}{0.45}\rangle$
$C_{2}$	$\langle \frac{\left[0.15,0.4\right]}{0.1},\frac{\left[0.25,0.55\right]}{0.35}\rangle$	$\langle \frac{\left[0.35,0.6\right]}{0.25},\frac{\left[0.15,0.35\right]}{0.55}\rangle$	$\langle \frac{\left[0.15,0.4\right]}{0.25},\frac{\left[0.25,0.55\right]}{0.35}\rangle$

**Table 6:** Expert *E*_1_ provides a decision matrix **M**^1^.

X	*ϑ*_1_	*ϑ*_2_	*ϑ*_3_	*ϑ*_4_
$C_{1}$	$\langle \frac{\left[0.3,0.45\right]}{0.3},\frac{\left[0.4,0.5\right]}{0.65}\rangle$	$\langle \frac{\left[0.3,0.55\right]}{0.2},\frac{\left[0.2,0.4\right]}{0.55}\rangle$	$\langle \frac{\left[0.3,0.55\right]}{0.4},\frac{\left[0.1,0.3\right]}{0.35}\rangle$	$\langle \frac{\left[0.25,0.4\right]}{0.6},\frac{\left[0.3,0.4\right]}{0.25}\rangle$
$C_{2}$	$\langle \frac{\left[0.1,0.5\right]}{0.2},\frac{\left[0.25,0.4\right]}{0.3}\rangle$	$\langle \frac{\left[0.2,0.45\right]}{0.35},\frac{\left[0.3,0.5\right]}{0.45}\rangle$	$\langle \frac{\left[0.35,0.7\right]}{0.5},\frac{\left[0.2,0.3\right]}{0.1}\rangle$	$\langle \frac{\left[0.2,0.6\right]}{0.3},\frac{\left[0.2,0.35\right]}{0.5}\rangle$
$C_{3}$	$\langle \frac{\left[0.2,0.5\right]}{0.55},\frac{\left[0.1,0.4\right]}{0.25}\rangle$	$\langle \frac{\left[0.15,0.5\right]}{0.4},\frac{\left[0.2,0.4\right]}{0.3}\rangle$	$\langle \frac{\left[0.2,0.4\right]}{0.25},\frac{\left[0.3,0.55\right]}{0.5}\rangle$	$\langle \frac{\left[0.3,0.65\right]}{0.6},\frac{\left[0.2,0.35\right]}{0.4}\rangle$
$C_{4}$	$\langle \frac{\left[0.3,0.55\right]}{0.7},\frac{\left[0.2,0.4\right]}{0.1}\rangle$,	$\langle \frac{\left[0.25,0.4\right]}{0.2},\frac{\left[0.3,0.5\right]}{0.25}\rangle$,	$\langle \frac{\left[0.3,0.5\right]}{0.45},\frac{\left[0.35,0.4\right]}{0.25}\rangle$,	$\langle \frac{\left[0.3,0.55\right]}{0.5},\frac{\left[0.1,0.4\right]}{0.45}\rangle$

It can be easily observed in Example 3.28 that $\cup_{R}$ and $\cap_{R}$ of two $\Omega_{E}$-sets may not be $\Omega_{E}$-set on X. In next result, we will show the criterion for $\cup_{R}$ and $\cap_{R}$ of two $\Omega_{E}$-sets is an $\Omega_{E}$-set on X.

Theorem 3.29 *Let*$\left(\Gamma_{1},A\right)=\langle \frac{{\overset{˜}{\sigma }}_{\Gamma_{1}}}{\sigma_{\Gamma_{1}}},\frac{{\overset{˜}{\varsigma }}_{\Gamma_{1}}}{\varsigma_{\Gamma_{1}}}\rangle$*and*$\left(\Gamma_{2},B\right)=\langle \frac{{\overset{˜}{\sigma }}_{\Gamma_{2}}}{\sigma_{\Gamma_{2}}},\frac{{\overset{˜}{\varsigma }}_{\Gamma_{2}}}{\varsigma_{\Gamma_{2}}}\rangle$*be two*$\Omega_{E}$*-sets on the universal set*X*such that*

$$\text{min}\{\text{max}\{\sigma_{\Gamma_{1}}^{L},\sigma_{\Gamma_{2}}^{U}\},\text{max}\{\sigma_{\Gamma_{1}}^{U},\sigma_{\Gamma_{2}}^{L}\}\}>\;(\sigma_{\Gamma_{1}}\wedge \sigma_{\Gamma_{2}})\;\geq \text{max}\{\text{min}\{\sigma_{\Gamma_{1}}^{L},\sigma_{\Gamma_{2}}^{U}\},\text{min}\{\sigma_{\Gamma_{1}}^{U},\sigma_{\Gamma_{2}}^{L}\}\}\text{and}\;\;\;\text{min}\{\text{max}\{\varsigma_{\Gamma_{1}}^{L},\varsigma_{\Gamma_{2}}^{U}\},\text{max}\{\varsigma_{\Gamma_{1}}^{U},\varsigma_{\Gamma_{2}}^{L}\}\}\geq \;(\varsigma_{\Gamma_{1}}\vee \varsigma_{\Gamma_{2}})\;>\text{max}\{\text{min}\{\varsigma_{\Gamma_{1}}^{L},\varsigma_{\Gamma_{2}}^{U}\},\text{min}\{\varsigma_{\Gamma_{1}}^{U},\varsigma_{\Gamma_{2}}^{L}\}\}$$

*then*$\left(\Gamma_{1},A\right)\cup_{R}\left(\Gamma_{2},B\right)$*is an*$\Omega_{E}$*-set on*X*.*

Proof For each x∈X, we take

$$\alpha_{x}=\text{min}\{\text{max}\{\sigma_{\Gamma_{1}}^{L},\sigma_{\Gamma_{2}}^{U}\},\text{max}\{\sigma_{\Gamma_{1}}^{U},\sigma_{\Gamma_{2}}^{L}\}\},\beta_{x}=\text{max}\{\text{min}\{\sigma_{\Gamma_{1}}^{L},\sigma_{\Gamma_{2}}^{U}\},\text{min}\{\sigma_{\Gamma_{1}}^{U},\sigma_{\Gamma_{2}}^{L}\}\},\alpha_{x}^{⁎}=\text{min}\{\text{max}\{\varsigma_{\Gamma_{1}}^{L},\varsigma_{\Gamma_{2}}^{U}\},\text{max}\{\varsigma_{\Gamma_{1}}^{U},\varsigma_{\Gamma_{2}}^{L}\}\},\beta_{x}^{⁎}=\text{max}\{\text{min}\{\varsigma_{\Gamma_{1}}^{L},\varsigma_{\Gamma_{2}}^{U}\},\text{min}\{\varsigma_{\Gamma_{1}}^{U},\varsigma_{\Gamma_{2}}^{L}\}\}.$$

Now $\alpha_{x}$ is one of the $\sigma_{\Gamma_{1}}^{L},\sigma_{\Gamma_{1}}^{U},\sigma_{\Gamma_{2}}^{L},\sigma_{\Gamma_{2}}^{U}$ and $\alpha_{x}^{⁎}$ is one of the $\varsigma_{\Gamma_{1}}^{L},\varsigma_{\Gamma_{1}}^{U},\varsigma_{\Gamma_{2}}^{L},\varsigma_{\Gamma_{2}}^{U}$. With out loss of generality we will consider only when $\alpha_{x}=\sigma_{\Gamma_{2}}^{L}$ and $\alpha_{x}^{⁎}=\varsigma_{\Gamma_{2}}^{L}$ or $\alpha_{x}=\sigma_{\Gamma_{2}}^{U}$ and $\alpha_{x}^{⁎}=\varsigma_{\Gamma_{2}}^{U}$. For the all remaining cases, the arguments are similar to these cases.

If $\alpha_{x}=\sigma_{\Gamma_{2}}^{L}$ and $\alpha_{x}^{⁎}=\varsigma_{\Gamma_{2}}^{L}$, then

$$\sigma_{\Gamma_{1}}^{L}\leq \sigma_{\Gamma_{1}}^{U}\leq \sigma_{\Gamma_{2}}^{L}\leq \sigma_{\Gamma_{2}}^{U},\varsigma_{\Gamma_{1}}^{L}\leq \varsigma_{\Gamma_{1}}^{U}\leq \varsigma_{\Gamma_{2}}^{L}\leq \varsigma_{\Gamma_{2}}^{U}.$$

So, $\beta_{x}=\sigma_{\Gamma_{1}}^{U}$ and $\beta_{x}^{⁎}=\varsigma_{\Gamma_{1}}^{U}$. Thus

$$\text{max}\{\sigma_{\Gamma_{1}}^{L},\sigma_{\Gamma_{2}}^{L}\}=\sigma_{\Gamma_{2}}^{L}=\alpha_{x}>(\sigma_{\Gamma_{1}}\wedge \sigma_{\Gamma_{2}})\;\;\;\;\;\;\text{and}\;\text{min}\{\varsigma_{\Gamma_{1}}^{U},\varsigma_{\Gamma_{2}}^{U}\}=\varsigma_{\Gamma_{1}}^{U}=\beta_{x}^{⁎}<(\varsigma_{\Gamma_{1}}\vee \varsigma_{\Gamma_{2}}).\text{So,}\;\;\;\;\;\;\;\;\;\;\;\;\;\;\;\;\;\;\;\;\;\;\;\;\;\;\;\;\;(\sigma_{\Gamma_{1}}\wedge \sigma_{\Gamma_{2}})\notin (\text{max}\{\sigma_{\Gamma_{1}}^{L},\sigma_{\Gamma_{2}}^{L}\},\text{max}\{\sigma_{\Gamma_{1}}^{U},\sigma_{\Gamma_{2}}^{U}\})=\text{rmax}\{{\overset{˜}{\sigma }}_{\Gamma_{1}},{\overset{˜}{\sigma }}_{\Gamma_{2}}\}\text{and}\;\;\;\;\;\;\;\;\;\;\;\;\;\;\;\;\;\;\;\;\;\;\;\;\;\;\;\;\;(\varsigma_{\Gamma_{1}}\vee \varsigma_{\Gamma_{2}})\notin (\text{min}\{\varsigma_{\Gamma_{1}}^{L},\varsigma_{\Gamma_{2}}^{L}\},\text{min}\{\varsigma_{\Gamma_{1}}^{U},\varsigma_{\Gamma_{2}}^{U}\})=\text{rmin}\{{\overset{˜}{\varsigma }}_{\Gamma_{1}},{\overset{˜}{\varsigma }}_{\Gamma_{2}}\}.$$

Thus, in this case $\left(\Gamma_{1},A\right)\cup_{R}\left(\Gamma_{2},B\right)$ is an $\Omega_{E}$-set on X.

If we take $\alpha_{x}=\sigma_{\Gamma_{2}}^{U}$ and $\alpha_{x}^{⁎}=\varsigma_{\Gamma_{2}}^{U}$, then

$$\sigma_{\Gamma_{1}}^{L}\leq \sigma_{\Gamma_{2}}^{U}\leq \sigma_{\Gamma_{1}}^{U}\;\;\;\;\;\;\;\;\text{and}\;\;\;\;\;\;\;\;\varsigma_{\Gamma_{1}}^{L}\leq \varsigma_{\Gamma_{2}}^{U}\leq \varsigma_{\Gamma_{1}}^{U}\;\text{so,}\;\;\;\;\;\;\beta_{x}=\text{max}\{\sigma_{\Gamma_{1}}^{L},\sigma_{\Gamma_{2}}^{L}\}\;\;\;\;\;\;\;\;\text{and}\;\;\;\;\;\;\;\;\beta_{x}^{⁎}=\text{max}\{\varsigma_{\Gamma_{1}}^{L},\varsigma_{\Gamma_{2}}^{L}\}.$$

Consider that $\beta_{x}=\sigma_{\Gamma_{1}}^{L}$ and $\beta_{x}^{⁎}=\varsigma_{\Gamma_{1}}^{L}$, then

$$\sigma_{\Gamma_{2}}^{L}\leq \sigma_{\Gamma_{1}}^{L}=\beta_{x}\leq (\sigma_{\Gamma_{1}}\wedge \sigma_{\Gamma_{2}})<\alpha_{x}=\sigma_{\Gamma_{2}}^{U}\leq \sigma_{\Gamma_{1}}^{U}\;\;\;\;\;\;\;\text{and}\;\varsigma_{\Gamma_{2}}^{L}\leq \;\;\varsigma_{\Gamma_{1}}^{L}=\beta_{x}^{⁎}<(\varsigma_{\Gamma_{1}}\vee \varsigma_{\Gamma_{2}})\leq \alpha_{x}^{⁎}=\varsigma_{\Gamma_{2}}^{U}\leq \varsigma_{\Gamma_{1}}^{U}.$$

We have following two cases.

$$\text{Case 1}\;\sigma_{\Gamma_{2}}^{L}\leq \sigma_{\Gamma_{1}}^{L}=\beta_{x}<(\sigma_{\Gamma_{1}}\wedge \sigma_{\Gamma_{2}})<\alpha_{x}=\sigma_{\Gamma_{2}}^{U}\leq \sigma_{\Gamma_{1}}^{U}\;\;\;\;\;\;\;\text{and}\;\;\varsigma_{\Gamma_{2}}^{L}\leq \;\;\varsigma_{\Gamma_{1}}^{L}=\beta_{x}^{⁎}<(\varsigma_{\Gamma_{1}}\vee \varsigma_{\Gamma_{2}})<\alpha_{x}^{⁎}=\varsigma_{\Gamma_{2}}^{U}\leq \varsigma_{\Gamma_{1}}^{U}\;\text{Case 2}\;\sigma_{\Gamma_{2}}^{L}\leq \sigma_{\Gamma_{1}}^{L}=\beta_{x}=(\sigma_{\Gamma_{1}}\wedge \sigma_{\Gamma_{2}})<\alpha_{x}=\sigma_{\Gamma_{2}}^{U}\leq \sigma_{\Gamma_{1}}^{U}\;\;\;\;\;\;\;\text{and}\;\;\varsigma_{\Gamma_{2}}^{L}\leq \;\;\varsigma_{\Gamma_{1}}^{L}=\beta_{x}^{⁎}<(\varsigma_{\Gamma_{1}}\vee \varsigma_{\Gamma_{2}})=\alpha_{x}^{⁎}=\varsigma_{\Gamma_{2}}^{U}\leq \varsigma_{\Gamma_{1}}^{U}.$$

Case 1 contradicts the fact that $\left(\Gamma_{1},A\right)$ and $\left(\Gamma_{2},B\right)$ are $\Omega_{E}$-sets on X. Case 2 implies that

$$(\sigma_{\Gamma_{1}}\wedge \sigma_{\Gamma_{2}})\notin (\text{max}\{\sigma_{\Gamma_{1}}^{L},\sigma_{\Gamma_{2}}^{L}\},\text{max}\{\sigma_{\Gamma_{1}}^{U},\sigma_{\Gamma_{2}}^{U}\})\;\;\;\;\;\;\;\;\;\;∵\;\;\;\;\;\;\;\text{max}\{\sigma_{\Gamma_{1}}^{L},\sigma_{\Gamma_{2}}^{L}\}=\sigma_{\Gamma_{1}}^{L}=(\sigma_{\Gamma_{1}}\wedge \sigma_{\Gamma_{2}})\text{and}\;\;\;(\varsigma_{\Gamma_{1}}\vee \varsigma_{\Gamma_{2}})\notin (\text{min}\{\varsigma_{\Gamma_{1}}^{L},\varsigma_{\Gamma_{2}}^{L}\},\text{min}\{\varsigma_{\Gamma_{1}}^{U},\varsigma_{\Gamma_{2}}^{U}\})\;\;\;\;\;\;\;\;\;\;∵\;\;\;\;\;\;\;\text{min}\{\varsigma_{\Gamma_{1}}^{U},\varsigma_{\Gamma_{2}}^{U}\}\;=\varsigma_{\Gamma_{2}}^{U}\;=(\varsigma_{\Gamma_{1}}\vee \varsigma_{\Gamma_{2}}).$$

We can obtain the similar result if we assume that $\beta_{x}=\sigma_{\Gamma_{1}}^{L},\beta_{x}^{⁎}=\varsigma_{\Gamma_{2}}^{L}\;\;\;\;\text{or}\;\;\;\;\beta_{x}=\sigma_{\Gamma_{2}}^{L},\beta_{x}^{⁎}=\varsigma_{\Gamma_{1}}^{L}\;\;\;\;\text{and}\;\;\;\;\beta_{x}=\sigma_{\Gamma_{1}}^{L},\beta_{x}^{⁎}=\varsigma_{\Gamma_{2}}^{L}$. Hence, $\left(\Gamma_{1},A\right)\cup_{R}\left(\Gamma_{2},B\right)$ is an $\Omega_{E}$-set on X. □

The following theorem can be easily verified and proved; therefore, we omit the details.

Theorem 3.30 *Let*$\left(\Gamma_{1},A\right)=\langle \frac{{\overset{˜}{\sigma }}_{\Gamma_{1}}}{\sigma_{\Gamma_{1}}},\frac{{\overset{˜}{\varsigma }}_{\Gamma_{1}}}{\varsigma_{\Gamma_{1}}}\rangle$*and*$\left(\Gamma_{2},B\right)=\langle \frac{{\overset{˜}{\sigma }}_{\Gamma_{2}}}{\sigma_{\Gamma_{2}}},\frac{{\overset{˜}{\varsigma }}_{\Gamma_{2}}}{\varsigma_{\Gamma_{2}}}\rangle$*be two*$\Omega_{E}$*-sets on the universal set*X*such that*

$$\text{min}\{\text{max}\{\sigma_{\Gamma_{1}}^{L},\sigma_{\Gamma_{2}}^{U}\},\text{max}\{\sigma_{\Gamma_{1}}^{U},\sigma_{\Gamma_{2}}^{L}\}\}\geq \;(\sigma_{\Gamma_{1}}\vee \sigma_{\Gamma_{2}})\;>\text{max}\{\text{min}\{\sigma_{\Gamma_{1}}^{L},\sigma_{\Gamma_{2}}^{U}\},\text{min}\{\sigma_{\Gamma_{1}}^{U},\sigma_{\Gamma_{2}}^{L}\}\}\text{and}\;\;\;\text{min}\{\text{max}\{\varsigma_{\Gamma_{1}}^{L},\varsigma_{\Gamma_{2}}^{U}\},\text{max}\{\varsigma_{\Gamma_{1}}^{U},\varsigma_{\Gamma_{2}}^{L}\}\}>\;(\varsigma_{\Gamma_{1}}\wedge \varsigma_{\Gamma_{2}})\;\geq \text{max}\{\text{min}\{\varsigma_{\Gamma_{1}}^{L},\varsigma_{\Gamma_{2}}^{U}\},\text{min}\{\varsigma_{\Gamma_{1}}^{U},\varsigma_{\Gamma_{2}}^{L}\}\}$$

*then*$\left(\Gamma_{1},A\right)\cap_{R}\left(\Gamma_{2},B\right)$*is an*$\Omega_{E}$*-set on*X*.*

Proof The proof is similar to Theorem 3.29; therefore, we omit the details. □

Theorem 3.31 *Let*$\left(\Gamma_{1},A\right)=\langle \frac{{\overset{˜}{\sigma }}_{\Gamma_{1}}}{\sigma_{\Gamma_{1}}},\frac{{\overset{˜}{\varsigma }}_{\Gamma_{1}}}{\varsigma_{\Gamma_{1}}}\rangle$*and*$\left(\Gamma_{2},B\right)=\langle \frac{{\overset{˜}{\sigma }}_{\Gamma_{2}}}{\sigma_{\Gamma_{2}}},\frac{{\overset{˜}{\varsigma }}_{\Gamma_{2}}}{\varsigma_{\Gamma_{2}}}\rangle$*be any two*$\Omega_{I}$*-sets on the universal set*X*such that*$\text{min}\{\sigma_{\Gamma_{1}},\sigma_{\Gamma_{2}}\}\leq \text{max}\{\sigma_{\Gamma_{1}}^{L},\sigma_{\Gamma_{2}}^{L}\}\;\text{and}\;\text{max}\{\sigma_{\Gamma_{1}},\sigma_{\Gamma_{2}}\}\geq \text{min}\{\varsigma_{\Gamma_{1}}^{U},\varsigma_{\Gamma_{2}}^{U}\}$*then*$\left(\Gamma_{1},A\right)\cup_{R}\left(\Gamma_{2},B\right)$*is*$\Omega_{E}$*-set on*X*.*

Theorem 3.32 *Let*$\left(\Gamma_{1},A\right)=\langle \frac{{\overset{˜}{\sigma }}_{\Gamma_{1}}}{\sigma_{\Gamma_{1}}},\frac{{\overset{˜}{\varsigma }}_{\Gamma_{1}}}{\varsigma_{\Gamma_{1}}}\rangle$*and*$\left(\Gamma_{2},B\right)=\langle \frac{{\overset{˜}{\sigma }}_{\Gamma_{2}}}{\sigma_{\Gamma_{2}}},\frac{{\overset{˜}{\varsigma }}_{\Gamma_{2}}}{\varsigma_{\Gamma_{2}}}\rangle$*be any two*$\Omega_{I}$*-sets on the universal set*X*such that*$\text{max}\{\sigma_{\Gamma_{1}},\sigma_{\Gamma_{2}}\}\geq \text{min}\{\sigma_{\Gamma_{1}}^{U},\sigma_{\Gamma_{2}}^{U}\}\;\text{and}\;\text{min}\{\sigma_{\Gamma_{1}},\sigma_{\Gamma_{2}}\}\leq \text{max}\{\varsigma_{\Gamma_{1}}^{L},\varsigma_{\Gamma_{2}}^{L}\}$*then*$\left(\Gamma_{1},A\right)\cap_{R}\left(\Gamma_{2},B\right)$*is*$\Omega_{E}$*-set on*X*.*

## 4 Application of Ω-set in MADM

This section describes the set-theoretic aggregation operations of a Ω-set that were applied in the design of a MADM recommendation model.

### 4.1 Problem description

Although thermal and hydroelectric energy sources are more expensive than other energy sources, they are used to produce electricity in some developing nations, such as Pakistan. Since alternative energy sources require slightly investment in the beginning, all types of customers choose to utilize them. Since solar panels are the most important kind of alternative energy in this context, their use in Pakistan has risen dramatically. Many clients from various sectors have made the switch to solar power systems. Homes, workplaces, farms, and industries all use it. These environmentally friendly solar systems ensure energy conservation, making them an intelligent option for consumers. Pakistan offers abundant sunlight throughout the year, making solar energy a sensible option for clean and renewable energy. On the market, there are multiple kinds of solar panels. Customers are very careful and concerned about their purchases because low-quality and inadequate solar panels are easily accessible. The process of choosing high-quality, long-lasting solar panels is difficult and involves several factors. Under the circumstances of an unclear algebraic setting, the MADM technique greatly assists decision-makers in choosing an appropriate solar panel product by taking into account relevant parameters. With the aid of some decision-makers, an algorithm (Algorithm 4.1) is suggested in the subsection that follows to help consumers buy a suitable solar panel product.

Algorithm 4.1 Let $X=\{C_{1},C_{2},...,C_{m}\}$ be the set of choices, $Z=\{Z_{1},Z_{2},...,Z_{n}\}$ be the set of attributes which has following sub attributes values $Z_{i}=\{e_{i1},e_{i2},...,e_{ix}\}$ for each 1≤i≤n,x∈{1,2,3,....} and $E=\{E_{1},E_{2},...,E_{k}\}$ be the set of experts. Suppose that $\vartheta =\{\vartheta_{1},\vartheta_{2},...,\vartheta_{j^{\prime }}\}$ is the set of *n*-tuples of $G=\prod_{i=1}^{n}Z_{i}$. Assume that each alternative $C_{i^{\prime }}\;(1\leq i^{\prime }\leq n)$ is observed by experts $E_{k}\;(1\leq k\leq K)$ with respect to each ϑj∈G using the Ω-sets. The suggested MADM algorithm's step-by-step growth is shown below.

1. Based on the evaluated values of the experts $E_{k}\;(1\leq k\leq K)$ in the form of Ω-sets $a_{ij}^{k}$, construct the decision matrices $M^{k}={\left(a_{ij}^{k}\right)}_{m\times n}$.
2. Apply the suggested operations provided in Definition 3.11, Definition 3.13 to calculate the aggregated decision matrix $M={\left(a_{ij}\right)}_{m\times n}$ where $a_{ij}=\underset{1\leq k\leq K}{{⋃}_{P}}a_{ij}^{k}$ or $a_{ij}=\underset{1\leq k\leq K}{{⋃}_{R}}a_{ij}^{k}$.
3. Using Definition 3.4, determine the score value for each of the $a_{ij}$ in the aggregated decision matrix **M**.
4. Determine the preferred value for every alternative $C_{i}\;(1\leq i\leq m)$ where $P(C_{i})=\sum_{i=1}^{m}\sum_{j=1}^{n}a_{ij}$
5. Create the ranking order of the alternatives using the preference value's non-increasing order.

Example 4.2 Mr. Smith is a renowned landlord with sizable farmland. Electricity and gas farming have grown more challenging due to increased bills. He has chosen to install a solar panel system instead of such utility systems because it is more cost-effective as well as beneficial. However, he is worried about the number of inferior local brands available, so he has hired a few professionals to help him choose a decent brand based on several qualities. So, he and the experts examined the technical details of several models available on the market. Consider the universe of discourse, $X=\{C_{1},C_{2},C_{3},C_{4}\}$ consisting of four different solar panel models manufactured by various companies. For this purchase, the set of experts is represented by $E=\{E_{1},E_{2},E_{3}\}$. After careful analysis of the literature [37], [48], [49], [50], [51], [52] and with the mutual understanding of the experts, attributes like rated power (pmax), cell size, wafer type, cell technology, and efficiency have been adopted for this evaluation. To make a sound decision, the respective sub-attributes of these attributes are $Z_{1}=\{e_{11}=744\;\text{W},e_{12}=730\;\text{W},e_{13}=700\;\text{W},e_{14}=625\;\text{W}\}$, $Z_{2}=\{e_{21}=210\;\text{mm},e_{22}=182\;\text{mm}\}$, $Z_{3}=\{e_{31}=N-typer,\;e_{32}=P-typer\}$, $Z_{4}=\{e_{41}=\text{Hetero junction (HJT)},e_{42}=\text{Tunnel-Oxide}\;\text{Passivating}\;\text{Contact (TOPCon )},e_{43}=\text{Mono PERC+}\;\text{(Passivated emitter}\;\&\;\text{rear cell) }\}$, and $Z_{5}=\{e_{51}=23.96\text{\%},e_{52}=23.5\text{\%},e_{53}=22.5\text{\%}\}$. To construct an Ω-set, the Cartesian product $Z=Z_{1}\times Z_{2}\times Z_{3}\times Z_{4}\times Z_{5}$ of disjoint attributive valued sets is determined, which consists of 144 sub-attributes valued tuples, however, for the sake of avoiding computational complexity and getting reliability, four tuples $\left(e_{11},e_{21},e_{31},e_{41},e_{51}\right)$, $\left(e_{12},e_{22},e_{32},e_{42},e_{53}\right)$, $\left(e_{13},e_{21},e_{32},e_{43},e_{52}\right)$ and $\left(e_{14},e_{22},e_{31},e_{41},e_{51}\right)$ have been chosen for further evaluation. These tuples are given the names as $\vartheta_{1}$, $\vartheta_{2}$, $\vartheta_{3}$ and $\vartheta_{4}$ respectively. Assume the expert $E_{k}\;(k=1,2,3)$ used Ω-set to evaluate each option $C_{i}(i=1,2,3,4)$ based on the criteria $\vartheta_{j}\;(j=1,2,3,4)$. Each expert collects the information in the form of Ω-set, in which IVIFS is the opinion of the dealer and the corresponding IFS is agreement as well as disagreement with IVIFS. We will proceed with the following steps:

**Step 1** The expertise of experts is used to generate the decision matrices $M^{1},M^{2},M^{3}$, as shown in Table 6, Table 7 and Table 8.

**Table 7:** Expert *E*_2_ provides a decision matrix **M**^2^.

X	*ϑ*_1_	*ϑ*_2_	*ϑ*_3_	*ϑ*_4_
$C_{1}$	$\langle \frac{\left[0.2,0.6\right]}{0.65},\frac{\left[0.1,0.25\right]}{0.3}\rangle$	$\langle \frac{\left[0.45,0.6\right]}{0.4},\frac{\left[0.4,0.55\right]}{0.35}\rangle$	$\langle \frac{\left[0.2,0.4\right]}{0.15},\frac{\left[0.25,0.5\right]}{0.4}\rangle$	$\langle \frac{\left[0.3,0.45\right]}{0.15},\frac{\left[0.2,0.45\right]}{0.6}\rangle$
$C_{2}$	$\langle \frac{\left[0.1,0.35\right]}{0.4},\frac{\left[0.2,0.45\right]}{0.5}\rangle$	$\langle \frac{\left[0.1,0.35\right]}{0.4},\frac{\left[0.4,0.55\right]}{0.35}\rangle$	$\langle \frac{\left[0.45,0.6\right]}{0.65},\frac{\left[0.1,0.3\right]}{0.3}\rangle$	$\langle \frac{\left[0.1,0.35\right]}{0.4},\frac{\left[0.4,0.55\right]}{0.35}\rangle$
$C_{3}$	$\langle \frac{\left[0.2,0.35\right]}{0.5},\frac{\left[0.4,0.65\right]}{0.1}\rangle$	$\langle \frac{\left[0.3,0.45\right]}{0.5},\frac{\left[0.1,0.5\right]}{0.2}\rangle$	$\langle \frac{\left[0.3,0.6\right]}{0.5},\frac{\left[0.1,0.3\right]}{0.25}\rangle$	$\langle \frac{\left[0.2,0.35\right]}{0.15},\frac{\left[0.4,0.65\right]}{0.7}\rangle$
$C_{4}$	$\langle \frac{\left[0.25,0.6\right]}{0.7},\frac{\left[0.1,0.35\right]}{0.25}\rangle$	$\langle \frac{\left[0.3,0.45\right]}{0.5},\frac{\left[0.15,0.3\right]}{0.4}\rangle$	$\langle \frac{\left[0.45,0.6\right]}{0.4},\frac{\left[0.1,0.3\right]}{0.35}\rangle$	$\langle \frac{\left[0.1,0.35\right]}{0.65},\frac{\left[0.4,0.55\right]}{0.3}\rangle$

**Table 8:** Expert *E*_3_ provides a decision matrix **M**^3^.

X	*ϑ*_1_	*ϑ*_2_	*ϑ*_3_	*ϑ*_4_
$C_{1}$	$\langle \frac{\left[0.3,0.45\right]}{0.4},\frac{\left[0.4,0.5\right]}{0.45}\rangle$	$\langle \frac{\left[0.2,0.3\right]}{0.5},\frac{\left[0.4,0.55\right]}{0.25}\rangle$	$\langle \frac{\left[0.2,0.6\right]}{0.5},\frac{\left[0.2,0.4\right]}{0.3}\rangle$	$\langle \frac{\left[0.25,0.6\right]}{0.5},\frac{\left[0.2,0.4\right]}{0.35}\rangle$
$C_{2}$	$\langle \frac{\left[0.1,0.35\right]}{0.6},\frac{\left[0.4,0.6\right]}{0.3}\rangle$	$\langle \frac{\left[0.4,0.6\right]}{0.3},\frac{\left[0.15,0.4\right]}{0.5}\rangle$	$\langle \frac{\left[0.3,0.6\right]}{0.5},\frac{\left[0.2,0.35\right]}{0.4}\rangle$	$\langle \frac{\left[0.1,0.4\right]}{0.3},\frac{\left[0.35,0.6\right]}{0.7}\rangle$
$C_{3}$	$\langle \frac{\left[0.25,0.5\right]}{0.5},\frac{\left[0.1,0.3\right]}{0.25}\rangle$	$\langle \frac{\left[0.15,0.4\right]}{0.1},\frac{\left[0.2,0.5\right]}{0.35}\rangle$	$\langle \frac{\left[0.3,0.6\right]}{0.7},\frac{\left[0.2,0.35\right]}{0.2}\rangle$	$\langle \frac{\left[0.15,0.4\right]}{0.2},\frac{\left[0.2,0.5\right]}{0.25}\rangle$
$C_{4}$	$\langle \frac{\left[0.2,0.4\right]}{0.3},\frac{\left[0.25,0.5\right]}{0.5}\rangle$	$\langle \frac{\left[0.3,0.5\right]}{0.25},\frac{\left[0.15,0.4\right]}{0.55}\rangle$	$\langle \frac{\left[0.15,0.4\right]}{0.25},\frac{\left[0.3,0.5\right]}{0.55}\rangle$	$\langle \frac{\left[0.4,0.6\right]}{0.5},\frac{\left[0.1,0.35\right]}{0.45}\rangle$
**Step 2** As mentioned in Definition 3.10, the suggested $\cup_{P}$ operation is used to build the aggregated decision matrix $M={\left(a_{ij}\right)}_{4\times 4}$ where $a_{ij}=\underset{1\leq k\leq K}{{⋃}_{P}}a_{ij}^{k}$. Table 9 displays the aggregated decision matrix **M**.

**Table 9:** Aggregated decision matrix **M** by using ∪_*P*_ operation.

X	*ϑ*_1_	*ϑ*_2_	*ϑ*_3_	*ϑ*_4_
$C_{1}$	$\langle \frac{\left[0.3,0.6\right]}{0.65},\frac{\left[0.1,0.25\right]}{0.3}\rangle$	$\langle \frac{\left[0.45,0.6\right]}{0.5},\frac{\left[0.2,0.4\right]}{0.25}\rangle$	$\langle \frac{\left[0.3,0.6\right]}{0.5},\frac{\left[0.1,0.3\right]}{0.3}\rangle$	$\langle \frac{\left[0.3,0.6\right]}{0.6},\frac{\left[0.2,0.4\right]}{0.25}\rangle$
$C_{2}$	$\langle \frac{\left[0.1,0.5\right]}{0.6},\frac{\left[0.2,0.4\right]}{0.3}\rangle$	$\langle \frac{\left[0.4,0.6\right]}{0.4},\frac{\left[0.15,0.4\right]}{0.35}\rangle$	$\langle \frac{\left[0.3,0.7\right]}{0.65},\frac{\left[0.1,0.3\right]}{0.1}\rangle$	$\langle \frac{\left[0.2,0.6\right]}{0.4},\frac{\left[0.2,0.35\right]}{0.35}\rangle$
$C_{3}$	$\langle \frac{\left[0.25,0.5\right]}{0.55},\frac{\left[0.1,0.3\right]}{0.1}\rangle$	$\langle \frac{\left[0.3,0.5\right]}{0.5},\frac{\left[0.1,0.4\right]}{0.2}\rangle$	$\langle \frac{\left[0.3,0.7\right]}{0.6},\frac{\left[0.1,0.3\right]}{0.2}\rangle$	$\langle \frac{\left[0.15,0.65\right]}{0.6},\frac{\left[0.2,0.35\right]}{0.25}\rangle$
$C_{4}$	$\langle \frac{\left[0.3,0.6\right]}{0.7},\frac{\left[0.1,0.35\right]}{0.1}\rangle$	$\langle \frac{\left[0.3,0.5\right]}{0.5},\frac{\left[0.15,0.3\right]}{0.25}\rangle$	$\langle \frac{\left[0.45,0.6\right]}{0.45},\frac{\left[0.1,0.3\right]}{0.25}\rangle$	$\langle \frac{\left[0.4,0.6\right]}{0.65},\frac{\left[0.1,0.35\right]}{0.3}\rangle$
**Step 3** The score value of each $a_{ij}$ in the aggregated decision matrix **M** will be determined by applying Definition 3.4. Table 10 displays the matrix of score values for all the elements of **M**.

**Table 10:** Score values of **M**.

X	*ϑ*_1_	*ϑ*_2_	*ϑ*_3_	*ϑ*_4_
$C_{1}$	0.3	0.2333	0.2333	0.2167
$C_{2}$	0.1	0.1667	0.3833	0.1
$C_{3}$	0.2667	0.2	0.3333	0.2
$C_{4}$	0.35	0.2	0.2833	0.3
**Step 4, 5** Calculate the preferred value of each 5-tuple sub parametric attributes $\vartheta_{j}\;(j=1,2,3,4)$ where $P(\vartheta_{j})=\max_{i=1}^{4}a_{ij}$ corresponding to each alternatives by using the $\cup_{P}$ operation are given below:
  $$P(\vartheta_{1})=0.35,P(\vartheta_{2})=0.2333,P(\vartheta_{3})=0.3833\text{ and }P(\vartheta_{4})=0.3.$$
  Based on the non-increasing order of their preference values of each 5-tuple sub-parametric attribute is ranked in the following order:
  $$P(\vartheta_{3})=0.3833\geq P(\vartheta_{1})=0.35\geq P(\vartheta_{4})=0.3\geq P(\vartheta_{2})=0.2333.$$

Similarly, the preferred value of each 5-tuple sub-parametric attributes $\vartheta_{j}\;(j=1,2,3,4)$ where $P(\vartheta_{j})=\max_{i=1}^{4}a_{ij}$ concerning each alternative by using the $\cup_{R}$ operation, is calculated and given in ranking order as follows:

$$P(\vartheta_{3})=0.2333\geq P(\vartheta_{1})=0.2\geq P(\vartheta_{4})=0.2\geq P(\vartheta_{2})=0.0833.$$

**Figure 1:**
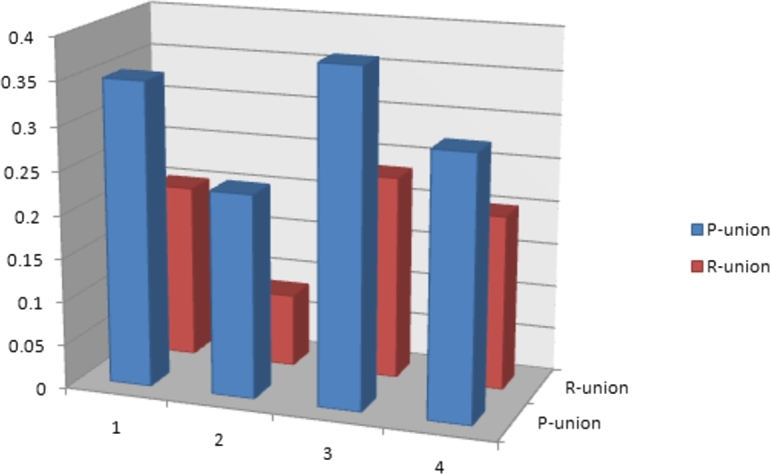
Ranking based on ∪_*P*_ and ∪_*R*_.

The robustness of the suggested method can be seen by the observation that the ranking order of sub-parametric attributes acquired with the aid of the $\cup_{P}$ operation and the $\cup_{R}$ operation is identical. Using the $\cap_{P}$ and $\cap_{R}$ processes described in Definition 3.11, Definition 3.13, we can readily observe that the sub-parametric attribute ranking order will result in the reverse order of the ranking orders acquired in the $\cup_{P}$ and $\cup_{R}$ operations, respectively (Fig. 1).

### 4.2 Comparison

It has not yet been explored how combinations of structures incorporating CIFS and HSS can be developed. This method can help increase the effectiveness and productivity of DM processes by dissecting each attribute into its parts, leading to a greater understanding of DM qualities. The Ω-set), a hybrid fuzzy structure, has been developed in recognition of a requirement for an adaptable analytical instrument that could be completely analyzed at the sub-attribute level. The combination of two membership functions: membership and non-membership, with two fuzzy intervals-membership and non-membership intervals that either include or lack both membership and non-membership functions, makes the CIFS a powerful contribution to FS theory. As a result, representing ambiguity and uncertainty in DM processes is made simpler. On the other hand, a sub-attribute analysis of attribute-based data from SS theory is the main emphasis of HSS theory. The Ω-set has been developed by combining these two frameworks to provide a precise and flexible approach to decision analysis. It is more adaptable as compared to existing ones as it generalizes them. To emphasize the advantages, Table 11 provides a comparison based on the structure in which the drawbacks of some relevant prior research are considered about the proposed framework.

**Table 11:** Structure based comparison.

Literature	Case study	Approach	Shortcomings
Rani et al. [37]	SPS	Pythagorean fuzzy DM	Two dimensional arrangement of IVIFS and IFS are missing.
Ihsan et al. [38]	SPS	FHSS with multi decisive settings	Two dimensional arrangement of IVIFS and IFS are missing.
Akram et al. [39]	SPS	Fermatean FSS with multi decisive settings	Two dimensional arrangement of IVIFS and IFS are missing.
Riaz et al. [46]	Renewable Energy Resources	Cubic bipolar fuzzy DM	Two dimensional arrangement of IVIFS and IFS are missing.
Suggested framework	SPS	Ω-set	A proper formulation has been provided to manage uncertainties effectively using two dimensional arrangement of IVIFS and IFS are missing.

Additionally, some notable advantages of this study are:

1. By promoting collaboration, the suggested context, the Ω-set, resolves the limitations of IFS and IFSS about the reliability of decision-makers opinions. At the same time, giving them specific ranges for these values improves their capacity to make well-informed and useful judgments.
2. In comparison to SS and IFSS, the approximate function of Ω-set is more flexible due to its multi-argument domain that takes into account the Cartesian product of attribute-valued non-overlapping sets. Its flexible range is cubic, allowing it to manage IFSS-related issues and facilitate well-informed DM.
3. The ability of Ω-set to capture expert judgments with both interval-valued and single-valued membership and non-membership grades improves flexibility and accuracy. This multifaceted approach enables more complex and in-depth evaluations, which makes it particularly useful in scenarios where precise analysis and DM require complex and multilayered data.

## 5 Conclusion

In this research effort, we presented a new hybrid structure combining a hypersoft set and a cubic intuitionistic fuzzy set called the Cubic intuitionistic fuzzy hypersoft set (Ω-set). We talked about some of its relevant features. We additionally discussed the $\Omega_{I}$-set and $\Omega_{E}$-sets, two more kinds of Ω-sets. Along with the relevant examples, the P-order, R-order, $\cup_{P}$, $\cup_{R}$, $\cap_{P}$, $\cap_{R}$, and several other helpful properties were also discussed. Furthermore, we established that $\Omega_{I}$-sets are also $\cup_{P}$, and $\cup_{R}$ of $\Omega_{I}$-sets. Further, this study established some conditions under which the $\cup_{P}$, $\cup_{R}$, $\cap_{P}$, and $\cap_{R}$ of two $\Omega_{E}$-sets are $\Omega_{I}$-sets. A few requirements for $\cup_{P}$, $\cup_{R}$, $\cap_{P}$, and $\cap_{R}$ of two $\Omega_{E}$-sets to be $\Omega_{E}$-sets were also given. A case study on the optimized evaluation of solar panels is presented, which validates a proposed MADM-based algorithm. In the case study, four different solar panel models are evaluated according to 14 sub-parameters, including rated power, cell size, wafer type, efficiency, and cell technology. The proposed framework is insufficient for situations where decision-makers want to provide expert opinions regarding other mathematical structures like picture fuzzy, spherical fuzzy, neutrosophic, or plithogenic cubic hypersoft settings.

## Declaration of generative AI and AI-assisted technologies in the writing process

During the preparation of this work the author(s) used chatGPT. After using this tool/service, the author(s) reviewed and edited the content as needed and take(s) full responsibility for the content of the publication.

## CRediT authorship contribution statement

**Muhammad Sajid:** Writing – original draft, Visualization, Methodology, Investigation, Formal analysis, Data curation, Conceptualization. **Khuram Ali Khan:** Writing – review & editing, Supervision, Methodology, Investigation, Formal analysis, Conceptualization. **Atiqe Ur Rahman:** Writing – original draft, Visualization, Methodology, Data curation, Conceptualization. **Sanaa A. Bajri:** Writing – review & editing, Software, Project administration, Funding acquisition, Data curation, Conceptualization. **Alhanouf Alburaikan:** Writing – review & editing, Software, Project administration, Funding acquisition, Data curation, Conceptualization. **Hamiden Abd El-Wahed Khalifa:** Writing – review & editing, Software, Project administration, Funding acquisition, Data curation, Conceptualization.

## Declaration of Competing Interest

The authors declare that they have no known competing financial interests or personal relationships that could have appeared to influence the work reported in this paper.

## Data Availability

Data will be made available on request.
